# Phylogeography Reveals Association between Swine Trade and the Spread of Porcine Epidemic Diarrhea Virus in China and across the World

**DOI:** 10.1093/molbev/msab364

**Published:** 2021-12-24

**Authors:** Wan-Ting He, Nena Bollen, Yi Xu, Jin Zhao, Simon Dellicour, Ziqing Yan, Wenjie Gong, Cheng Zhang, Letian Zhang, Meng Lu, Alexander Lai, Marc A Suchard, Xiang Ji, Changchun Tu, Philippe Lemey, Guy Baele, Shuo Su

**Affiliations:** 1 Jiangsu Engineering Laboratory of Animal Immunology, Institute of Immunology, College of Veterinary Medicine, Academy for Advanced Interdisciplinary Studies, Nanjing Agricultural University, Nanjing, China; 2 Department of Microbiology, Immunology and Transplantation, Rega Institute, KU Leuven, Leuven, Belgium; 3 China Animal Disease Control Center, Ministry of Agriculture, Beijing, China; 4 Spatial Epidemiology Lab (SpELL), Université Libre de Bruxelles, Bruxelles, Belgium; 5 Key Laboratory of Jilin Province for Zoonosis Prevention and Control, Institute of Military Veterinary, Academy of Military Medical Sciences, Changchun, Jilin, China; 6 School of Science, Technology, Engineering, and Mathematics, Kentucky State University, Frankfort, KY, USA; 7 Department of Biostatistics, Fielding School of Public Health, University of California Los Angeles, Los Angeles, CA, USA; 8 Department of Biomathematics, David Geffen School of Medicine, University of California Los Angeles, Los Angeles, CA, USA; 9 Department of Human Genetics, David Geffen School of Medicine, University of California Los Angeles, Los Angeles, CA, USA; 10 Department of Mathematics, School of Science & Engineering, Tulane University, New Orleans, LA, USA

**Keywords:** porcine epidemic diarrhea virus, Coronaviridae, phylogeography, Bayesian inference, generalized linear model, BEAST

## Abstract

The ongoing SARS (severe acute respiratory syndrome)-CoV (coronavirus)-2 pandemic has exposed major gaps in our knowledge on the origin, ecology, evolution, and spread of animal coronaviruses. Porcine epidemic diarrhea virus (PEDV) is a member of the genus Alphacoronavirus in the family Coronaviridae that may have originated from bats and leads to significant hazards and widespread epidemics in the swine population. The role of local and global trade of live swine and swine-related products in disseminating PEDV remains unclear, especially in developing countries with complex swine production systems. Here, we undertake an in-depth phylogeographic analysis of PEDV sequence data (including 247 newly sequenced samples) and employ an extension of this inference framework that enables formally testing the contribution of a range of predictor variables to the geographic spread of PEDV. Within China, the provinces of Guangdong and Henan were identified as primary hubs for the spread of PEDV, for which we estimate live swine trade to play a very important role. On a global scale, the United States and China maintain the highest number of PEDV lineages. We estimate that, after an initial introduction out of China, the United States acted as an important source of PEDV introductions into Japan, Korea, China, and Mexico. Live swine trade also explains the dispersal of PEDV on a global scale. Given the increasingly global trade of live swine, our findings have important implications for designing prevention and containment measures to combat a wide range of livestock coronaviruses.

## Introduction

Coronaviruses are single-stranded, positive-sense RNA viruses that cause subclinical as well as respiratory and gastrointestinal diseases in humans, other mammals, and birds (Brian and Baric 2005). The host range switches of coronaviruses (CoVs) from wildlife to humans have resulted in several novel diseases with high fatality rate, such as severe acute respiratory syndrome (SARS), Middle East respiratory syndrome (MERS), and the recent outbreak of SARS-CoV-2 (Drosten et al. 2003; Zaki et al. 2012; Sun et al. 2020). Of note, SARS-CoV-2 is the most widespread and impactful human infectious disease since the start of the 21st century (Drosten et al. 2003; Graham and Baric 2020). As of November 8, 2021, there have been over 250 million confirmed cases and 5 million deaths reported worldwide (https://www.who.int/data#reports). Despite likely having originated in bats, SARS-CoV and MERS-CoV have infected humans via an intermediate host rather than through direct infection from bats, and this may also be the case for SARS-CoV-2 (Guan et al. 2003; Lam et al. 2020; Zhou et al. 2020). Currently, the number of known intermediate hosts involved in the transmission of bat-origin coronaviruses to humans is limited. Compared with many other species, swine are in frequent contact with both humans and other animals such as wildlife, livestock, stray cats and dogs, and aquatic birds, which increases in theory the chance of cross-species transmission (Sabir et al. 2016; Li et al. 2018; He et al. 2019). Therefore, monitoring and understanding the evolution and transmission of porcine coronavirus in swine populations can not only help the swine breeding industry, but also further prevent and control potential public health threats.

Porcine epidemic diarrhea virus (PEDV) is a likely bat-associated *Alphacoronavirus* that causes porcine epidemic diarrhea (PED) (Huang et al. 2013). The main clinical syndrome is characterized by acute watery diarrhea, vomiting, and dehydration. In swine, the mortality rate can often reach 100% (Wood 1977; Coussement et al. 1982; Chen et al. 2011). In the past 30 years (from 1984 to early 2010), there has been sporadic circulation of PEDV in the swine population in China and worldwide (Sun et al. 2015; Wang et al. 2016). However, a larger PEDV outbreak occurred in southern China in late 2010, later spreading to other Chinese provinces (Sun et al. 2015; Wang et al. 2016). The identification and sequencing of PEDV strains in this outbreak showed the emergence of a new variant of PEDV in China (Chen et al. 2012), further contributing to an overall high diversity of PEDV variants in China (Chen et al. 2019; Su et al. 2020; Tan et al. 2020). However, compared with classic strains such as CV777 (G1 genogroup, subgroup G1a), this PEDV variant forms an independent lineage (G2 genogroup). Due to the genetic differences between the G1 and G2 genogroups, the commercially available CV777 inactivated vaccine and the DR13 attenuated live vaccine do not provide effective protection for PEDV variants, making prevention and control difficult to achieve (Li et al. 2012; Sun et al. 2012; Wang et al. 2013). PEDV G2 quickly became the dominant strain worldwide and currently circulates in swine farms in Asia, Europe, and North America causing enormous economic losses to the swine industry and posing a potential threat to public health (Huang et al. 2013).

Phylogeographic inference enables the reconstruction of the geographic dispersion of a virus through time, based upon the sampling times and sampling locations associated with the collected sequence data (Lemey et al. 2009, 2010). Different extensions have been developed to assess the impact of potential predictors, in the form of ecological and environmental data, on the geographic spread of viruses (Lemey et al. 2014; Dellicour et al. 2016). Certain phylogeographic studies have pointed toward live animal trade as an important source of viral spread. For example, the global spread of swine influenza A viruses was mainly influenced by live swine trade between countries (Nelson et al. 2015), whereas other studies have identified the important role of live poultry trade (Yang et al. 2020). Another study, focusing on the spread of type 2 porcine reproductive and respiratory syndrome virus (PRRSV) in North America, found a unidirectional PRRSV flow from Canada to the United States through live swine trade. After its introduction, and impacted by landscape structure, this variant rapidly expanded in genetic diversity and geographic distribution (Shi et al. 2013). These studies illustrate the potential of phylogenetic and phylogeographic inference for identifying reservoir species, sources of infection, the overall ancestral history of spread and the (ecological and/or environmental) factors that impact that spread (Holmes 2008).

China has seen the emergence of the highest number of livestock and poultry diseases in the world, and (re-)emerging diseases continue to occur (Zhai et al. 2014; Gu et al. 2017; Guo et al. 2018; Li et al. 2018; Dixon et al. 2019). China also hosts the largest swine population in the world, and while live swine transportation between provinces is frequent, the role of transportation and stocking density in spreading swine-borne viruses nationwide is still largely unclear (Nelson et al. 2015; Baele et al. 2018). For the purpose of this study, we were granted access to previously inaccessible between-province trade data, providing a unique opportunity to study transmission dynamics within China in detail. We composed an initial data set of over 2,000 sequences, including 247 new sequences extracted over the past 3 years and all publicly available PEDV sequences on GenBank, which we then ran through a rigorous data-selection process. We employed Bayesian phylogeographic inference (Lemey et al. 2009) using discrete location data (provinces within China and countries from across the world) in order to infer the evolutionary and geographical history of PEDV within China and on a global scale. We made use of an extension of this model—in the form of a generalized linear model (GLM)—that enables us to test the contribution of a collection of potential predictors of viral spread. To the best of our knowledge, our work constitutes the first study conducted on the impact of live swine trade on the geographic dispersal of swine-borne viruses in China. Furthermore, given the propensity of interspecies transmission by coronaviruses, and their tendency for recombination, our unique approach allowed us to bridge the knowledge gap on emergence of animal coronaviruses with cross-regional transmissions. This methodology also provides a framework for assessing risks of infectious diseases in livestock associated with patterns of live animal movement and environmental factors.

## Results

### Phylogeographic Reconstruction and Drivers of PEDV Spread within China

**Fig. 1.**
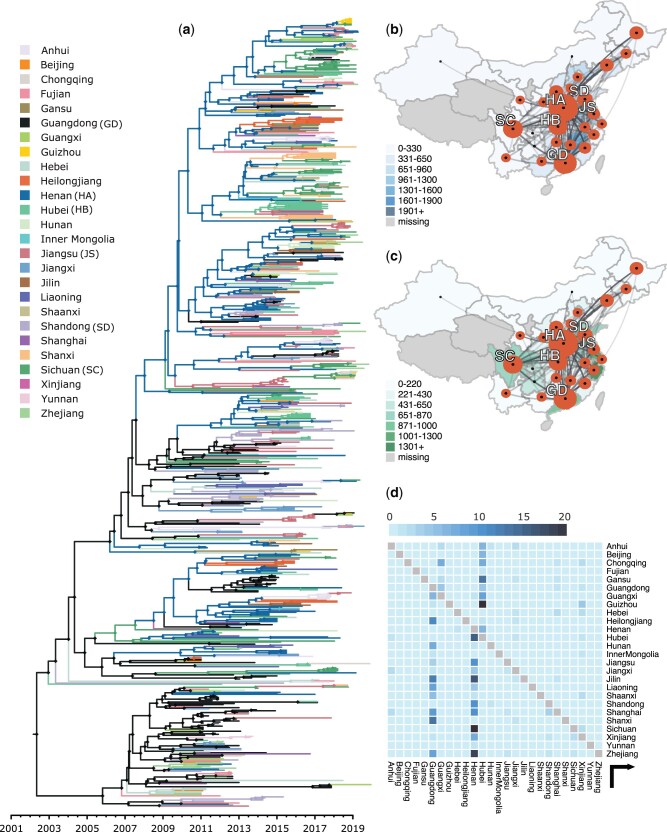
PEDV phylogeographic reconstruction within China. (*a*) Maximum clade credibility tree with ancestral nodes and branches colored according to estimated (province) location, depicting the spread of PEDV within China. The origin of the PEDV G2 epidemic is estimated to have started in July of 2003 (uncertainty ranges from March 2002 to May 2005) with an introduction into Guangdong (GD) (posterior support of 0.91), followed by multiple introductions into Henan, which then started seeding early introductions into Sichuan (SC) and Xinjiang after roughly 2 years. As of 2007, PEDV started spreading from Guangdong into Henan (HA) on multiple occasions and frequent introductions from Henan back into Guangdong can be observed throughout the past decade. Geographic spread of PEDV within China in the context of live swine trade intensity in 2017, as measured by: (*b*) import of live swine per province; (*c*) export of live swine per province; the size of the polygons in each province are proportional to the number of tree lineages that maintain that location, thereby capturing the absolute and relative intensity of spread. A larger polygon indicates a larger number of tree branches at that location through time. Westward movements are depicted by lines with an upward curvature, whereas eastward movements are depicted by lines with a downward curvature. The provinces of Henan and Guangdong act as the main hubs for the spread of PEDV within China, with Henan being the largest importer of live swine while barely exporting any. Henan and Jiangsu (JS) provinces show the highest total trade (import and export) levels, but Jiangsu province ranks only fifth in terms of the number of PEDV branches it maintains. (*d*) Estimated number of Markov jumps between provinces, showing Henan, Guangdong, and Hubei to be the main location causing introductions into other provinces.

Phylogenetic analysis revealed that long-distance movement of PEDV genotype G2 between provinces has occurred continuously in swine since 2010. Figure 1a shows the reconstructed spread of PEDV within China during the past two decades by means of the estimated MCC tree, based on the discrete phylogeographic inference with GLM parametrization. The results indicate an origin in Guangdong (posterior support of 0.91). Shortly after this estimated first occurrence in mainland China, a single sequence from both Shandong and Hubei branches off from an otherwise fully Guangdong-located backbone of the phylogenetic tree. However, the former is weakly supported (posterior support of 0.45), and we do not find these branches to be convincing evidence of an early introduction of PEDV into Shandong and Hubei. Subsequently, multiple introductions are estimated to have occurred from Guangdong into Henan, from which point onward Henan acts as the source of introductions into multiple provinces such as Sichuan, Liaoning, Shanxi, and Jiangsu. Guangdong itself acts as the main source of PEDV introductions into Zhejiang, Jiangxi, and Shandong. As displayed in figure 1a, PEDV spread from Guangdong into Henan on multiple occasions and frequent introductions from Henan back into Guangdong can be observed throughout the past 15 years. These results can also be seen in the Markov jumps plot in figure 1d (see supplementary table S2, Supplementary Material online, for the complete Markov jump matrix). Here, we see that most of the estimated transitions originated in Guangdong, Henan, and Hubei. Most of the introductions from Hubei are found near the tips of the phylogeny, indicating that this location might have acted as a tertiary hub later in the epidemic. From its inferred time of origin, the genetic diversity of PEDV quickly increased until reaching its peak in 2015 (supplementary fig. S7, Supplementary Material online), after which a steady decline can be observed. This decline gained momentum when ASFV appeared at the end of 2018, owing to the decrease of swine trade and number of pigs being bred.

As mentioned before, a key interest of this study is reconstructing the geographic dispersal and exploring the factors influencing the spread of PEDV within China. We considered various environmental, economic, and demographic variables at province level that might have an impact on the dissemination of PEDV (we refer to the Supplementary Material online for a list of all variables, their detailed descriptions and their sources, as well as to supplementary fig. S6, Supplementary Material online, showing the correlation matrix between variables). It is suspected that live animal trade might influence the spread of disease in farm animals (Nelson et al. 2015; Yang et al. 2020). Figure 1b and *c* explores the impact of trade by superimposing the number of imported live swine into each province and exported live swine by each province onto the spread of PEDV. Although Guangdong province imports almost no live swine, it exports a substantial amount and forms one of the main hubs for the spread of PEDV together with the province of Henan, which imports the most live swine in all of China and is estimated to act as a connecting hub for the spread of PEDV to many other provinces. We estimate Guangdong to have acted as a hub for dispersal to provinces within Southeast China and East China, that is, those provinces that are relatively close geographically, whereas Henan is connected to most other provinces across all of China and is estimated to have spread PEDV across longer distances. Finally, Hubei was estimated to have caused new introductions into nearby provinces, mostly to the west of the country. Although Jiangsu and Zhejiang are important provinces for the trade of live swine, they did not rank among the most important hubs for the spread of PEDV within China.

**Fig. 2.**
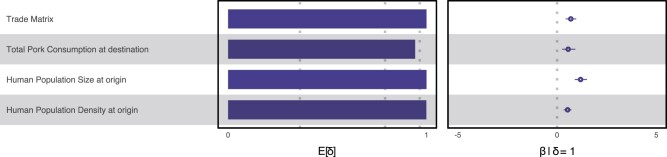
The support and contribution of PEDV diffusion predictors among 26 Chinese provinces when using live swine trade data from 2017. Among 34 predictors being considered, four were estimated to have a very strong impact on the spread of PEDV between provinces: live swine trade between provinces, pork consumption at destination, human population size at origin and human population density at origin. Support for each predictor is represented by an inclusion probability that is estimated as the posterior expectation for the indicator variable associated with each predictor (E[δ]). Indicator expectations corresponding to Bayes factor support values of 3, 20, and 150 are represented by a dotted vertical in this bar plot. The contribution of each predictor is represented by the mean and credible intervals of the GLM coefficients (β) on a log scale conditional on the predictor being included in the model (β|δ = 1). Only predictors whose credible interval excluded zero are shown.

Further variables of interest were those related to swine production and consumption, such as the price of swine feed, the amount of swine feed produced, the price of live swine and of swine meat, the number of swine slaughtered, the amount of swine meat consumed, and the total amount of food consumed. Another group of variables were those related to human demography and the economic impact of the province. These variables include total population, urban population density, and GDP for each region. Finally, we also considered a few geographical variables related to temperature, precipitation and vapor pressure, elevation, and the great-circle distance between provinces. We were able to recover very strong support for the contribution of live swine trade to the spread of PEDV within China (fig. 2; supplementary fig. S8, Supplementary Material online, shows the results for all variables). The variables based on the residuals of the regression for sample size against case counts were not found to be significant. The positive mean conditional effect size of live swine trade implies that transition rates between origin and destination provinces increase with increased live swine trade between those provinces. We also found strong support for the contribution of human population size and human population density at the province of origin to the spread of PEDV. Both mean conditional effect sizes are positive, indicating that transition rates between provinces increase with larger positive values for these predictors. Studies have highlighted the complexities of the wildlife–livestock–human interface. Urbanization can have drastic influences in the transmission dynamics of a disease (Hassell et al. 2017). Note that pork consumption, although estimated to have strong support for contributing to the spread of PEDV, probably does not directly influence dispersal; this factor has a positive correlation with (respectively) slaughterhouse contamination and frequency of vehicle transport between slaughterhouse and the swine farm (Lowe et al. 2014; Dee et al. 2016).

### Phylogeographic Reconstruction and Drivers of Global PEDV G2 Spread

**Fig. 3.**
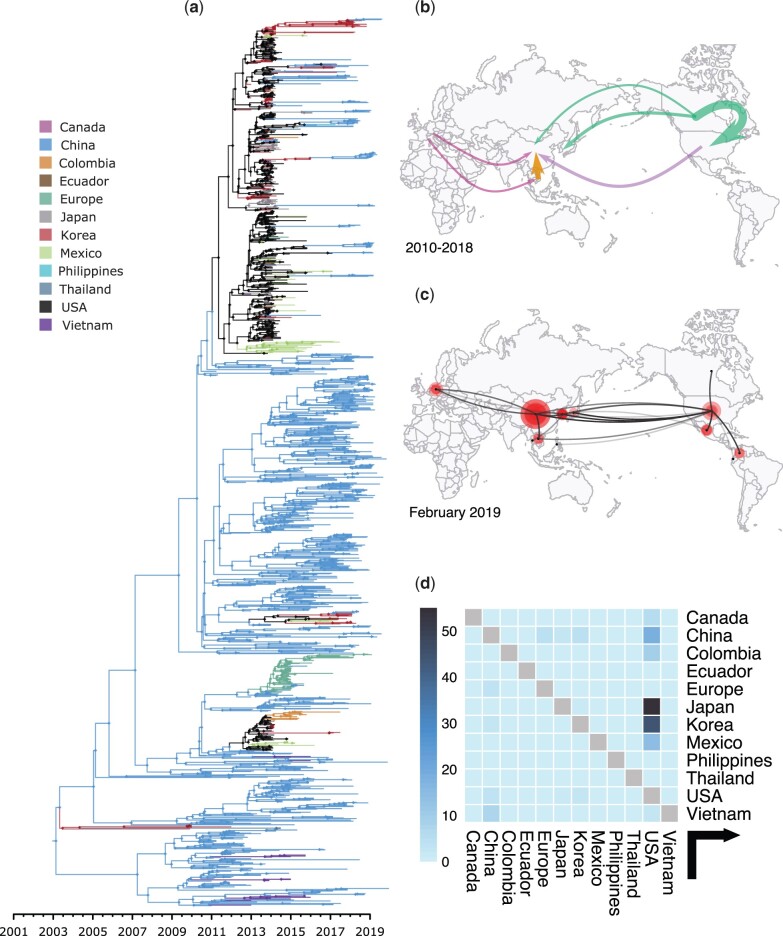
PEDV phylogeographic reconstruction across the world. (*a*) Maximum clade credibility tree with annotated countries on the ancestral nodes, depicting the global spread of PEDV G2. The PEDV epidemic is estimated to have started in China toward the start of 2003 (with a wide 95% HPD that ranges from September 1996 to July 2005), but only started gaining momentum after 2008. Many of the major clades are only poorly supported preventing strong conclusions concerning the timing of long-range introduction events. (*b*) Visual representation of the main live swine trade routes in 2010–2018 show that by far the most live swine are transported from Canada to the United States and internally within Europe (not shown), with much smaller levels of intercontinental trade occurring. Only trade routes transporting more than 3,000 pigs per year are shown. Although live swine are imported into China from across the world, China is not a major exporter of live swine. (*c*) Reconstructed spread of PEDV using spreaD3 (Bielejec et al. 2016) shows the estimated number of lineages maintained in each country (as indicated by the size of the polygons) and reconstructed transmissions between countries/regions. (*d*) Estimated number of Markov jumps between the countries/regions shows a large number of introductions events from the United States to mostly (and in that order) Japan, Korea, China, and Mexico. As China plays a small role in terms of live swine export, only a few Markov jumps from China to Vietnam and Europe are estimated, coming in at less than 10% of the jumps from the United States to Japan.

Figure 3a shows our global phylogeographic reconstruction by means of the estimated MCC tree. We note that posterior support values for many of the major clades were quite poor, indicating some uncertainty about the timing of introduction events. Based on the phylogeographic reconstruction, the United States and China maintain the highest number of PEDV lineages. Figure 3 shows that after an initial introduction from China, the United States acted as the main source of PEDV G2 introduction events (quantified by the estimated Markov jumps) into South Korea, Japan, China, and Mexico. We also estimate a few PEDV transmission events from China to Vietnam and Europe, as well as few PEDV transmission events from Europe into China. We visualize the main live swine trade routes between 2010 and 2018 in figure 3b, the estimated number of PEDV linages maintained in the different countries/regions in our data set in figure 3c, and the estimated number of Markov jumps between those countries/regions in figure 3d (see supplementary table S3, Supplementary Material online, for the complete Markov jump matrix). We estimated the highest number of transition events to have originated out of the United States, mostly to Japan (54), Korea (44), China (19), and Mexico (14). There were only a few introductions that originated out of China, the only notable numbers being to Vietnam (7), Europe (4), and the United States (3). Figure 3b shows that by far the largest numbers of live swine are being exported out of Canada to the United States (10 million pigs per year on average between 2010 and 2018). China is neither a big exporter nor importer of live pigs, with its main trade relationship occurring with Vietnam, from which it imported 800,000 pigs per year on average between 2010 and 2018 (see also supplementary table S1, Supplementary Material online). Other international but smaller trade flows are found to occur from Europe to China and Vietnam, from Vietnam to China, and from Canada to South Korea. Despite neither the United States nor China being large exporters, PEDV G2 is mostly found in China and the United States—as indicated through the estimated number of lineages maintained in these countries, shown in figure 3c—both countries play different but crucial roles in the dispersal history of PEDV G2.

In studying the global spread of PEDV between these 12 countries/regions, we consider several factors that potentially influence this spread. Besides a live swine trade matrix (see supplementary figs. S9 and S10, Supplementary Material online, with the former aggregating all European countries), we also consider total export and import of live swine, as well as GDP and the amount of live swine stocks in the country. Finally, as sampling sizes are expected to have an impact on the number of among-country transition events, we considered origin and destination sample sizes as separate predictors in our GLM. We report fairly strong correlation between these different predictors (supplementary fig. S11, Supplementary Material online). These predictors are similar to those in used in a global swine influenza study (Nelson et al. 2015). For a more detailed explanation of these variables and their sources, we refer to the Supplementary Material online. From its inferred time of origin, the genetic diversity of PEDV on a global scale quickly increased (supplementary fig. S12, Supplementary Material online). After 2015, a steady decline begins, which gained momentum toward 2017 and 2018, when ASFV appeared in different countries, resulting in a drastic decrease and temporary halt of swine trade.

**Fig. 4.**
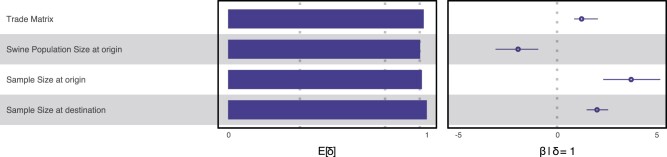
The support and contribution of PEDV diffusion predictors among 12 countries. Among the predictors being considered, four were estimated to have a very strong impact on the global spread of PEDV: live swine trade between provinces, sample size at origin, sample size at destination, and swine population size at destination. Support for each predictor is represented by an inclusion probability that is estimated as the posterior expectation for the indicator variable associated with each predictor (E[δ]). Indicator expectations corresponding to Bayes factor support values of 3, 20, and 150 are represented by a dotted vertical in this bar plot. The contribution of each predictor is represented by the mean and credible intervals of the GLM coefficients (β) on a log scale conditional on the predictor being included in the model (β|δ = 1). We note that there is a high degree of correlation between some of these variables including sample size (see supplementary fig. S11, Supplementary Material online). As sampling sizes are expected to have an impact on the number of location transitions, we considered origin and destination sample sizes as separate predictors in our GLM. However, even when taking into account sampling biases, we still found support for other factors in addition to sampling size predictors, which may suggest that they are robust (Lemey et al. 2014). Furthermore, including or excluding the sample sizes in the GLM analysis had minimal to no effect on the geographic reconstruction, further indicating robustness of the results. Only predictors whose credible interval excluded zero are shown.

We are able to recover with very strong support the positive contribution of live swine trade to the global spread of PEDV, along with very strong support for swine population size at the origin country, as well as sample size at origin and destination country (fig. 4; supplementary fig. S13, Supplementary Material online, shows the results for all variables). Importantly, the positive mean conditional effect size of live swine trade implies that transition rates between origin and destination countries increase with increased live swine trade between those countries. The negative mean conditional effect size of swine population size at origin country implies that countries with a large swine population are actually less involved with spreading PEDV. Although this might seem counter intuitive at first glance, we should note that for China, which has the largest swine population in the world (over 450 million swine), we only estimated very few Markov jumps into Europe and Mexico. The United States on the other hand has a smaller swine population (nearly 70 million swine) but is estimated to have seeded multiple PEDV introductions into Japan, Korean, China, Mexico, and to a lesser extent Colombia and Canada. We note the low number of inferred transmission events (as estimated through Markov jumps) from Canada to the United States, despite a high number of live swine being exported from Canada into the United States. Unfortunately, only six Canadian sequences could be included in our data set, limiting the number of transition events into Canada that can be estimated from any other country. We also note that the important predictors that contribute to the global PEDV spread are similar but not identical to those found in a previously published global swine influenza A (swIAV) study (Nelson et al. 2015). Although those authors also found very strong support for the contribution of live swine trade to the spread of swIAV, they did not report a strong contribution of swine population size, although they did find strong supports for the contribution of swine population size change, as well as positive support for sample size at the destination country.

## Discussion

Prevention and control of animal coronaviruses in China and across the world depend critically on a deep understanding of how these viruses spread and their mode of transmission across geographic and national boundaries. In this study, we assembled the largest collection of available PEDV sequences, including 247 newly sequenced viruses, and uniquely collated them with previously inaccessible swine trade data. By doing so, we were able to test and quantify a range of potential predictive variables for the spatial spread of emerging PEDV G2 both at local (China) and at global scale. In an increasingly globalized world, animal coronaviruses such as PEDV have ample opportunities to spread between countries and continents. As we have shown, there is international and even intercontinental trade of live swine between countries with millions of pigs being transported worldwide. Meanwhile, China hosts the largest swine population in the world with over 450 million heads. With such a large population, the probability of new strains of viruses emerging increases. Studying the dissemination patterns of PEDV on both a local and global scale offers key insights into the drivers of local and global PEDV spread and the main regions/countries that contribute to the emergence of PEDV in new territories.

We found that the spread of PEDV within China is strongly driven by live swine trade between provinces, with Guangdong and Henan acting as the main hubs of viral spread, seeding PEDV introductions into the other provinces, whereas Hubei province might have acted as a tertiary hub later in the epidemic, which mostly contributed to spread toward the west of the country. Our results provide further insights into large-scale swine viral disease outbreaks in China and help to identify key regions (as hot spots) that can be targeted to mitigate the progress of an epizootic epidemic. In addition, feed pollution (indirectly related) and pork consumption (leading to indirect abattoir pollution and vehicle pollution) may also be important factors contributing to the spread of PEDV in China, and these factors have previously been reported as influencing the spread of PEDV (Lowe et al. 2014; Dee et al. 2016). Our study also accurately reconstructed the early transmission of PEDV G2 from Guangdong to Henan in China, and the subsequent rapid spread to the northeast and southwest China such as to Sichuan province, an early long-distance movement which was most likely caused by live swine transportation. This finding was confirmed by the wider geographic spread of PEDV G2 in the Chinese Mainland from 2010 to 2013 (Sun et al. 2015). Given that Guangdong and Henan are the most intensely sampled provinces in our data set, we conducted a sensitivity analysis in order to ascertain a possible effect of unequal sampling on our inferred origin of the PEDV G2 epidemic within China. To this end, we performed a subsampling analysis in which we limited the number of sequences per province to at most 60 sequences. In practice, we randomly removed 59 sequences from Guangdong, 31 Henan, and 12 from Sichuan. This subsampling procedure results in five provinces having nearly equal sequence counts, reducing any artificial preference for Henan or Guangdong (the provinces with the highest number of sequences in our data set) to act as source provinces (above the other three provinces). The ensuing phylogeographic analysis yielded the same backbone structure of our location-annotated MCC tree (supplementary fig. S14, Supplementary Material online), again pointing to Guangdong and Henan as the key hubs for the spread of PEDV in China. The inferred Markov jump counts (supplementary fig. S15, Supplementary Material online) out of Guangdong but also out of Hubei are actually higher than in the full data set, whereas those out of Henan are lower compared with the full data set. This confirms our assessment that Guangdong and Henan play an important role as source locations, offering insights into where prevention and control efforts could be targeted to disrupt the movement of live swine to impact the spread of PEDV within China.

On a global scale, the United States and China maintain the highest number of PEDV lineages. We estimate that, after an initial introduction out of China, the United States acted as an important source of PEDV introductions into Japan, Korea, China, and Mexico. Most of the Asian and American introduction events came from the United States (as can be seen in fig. 3), which suggests the need for more intensified surveillance efforts on the export of live pigs, pork products, and feed from the United States to Latin America and Asia. Note that multiple studies have highlighted the genetic similarity between multiple outbreaks and the outbreak in the United States, characterizing these new strains as “US-like” (Lee and Lee 2014; Van Diep et al. 2018; Sung et al. 2019; Chen et al. 2021). China is not a major exporter of live swine, as reflected in our results: we estimated only a few PEDV transmission events from China, mostly to Vietnam and Europe. We also observe some introduction events back into China (from the United States, but also a few from Korea and Japan), further illustrating the interconnectedness of the global spread of PEDV.

We performed a state-of-the-art Bayesian discrete phylogeographic inference with its popular extension in the form of a GLM to test the contribution of a large number of predictors to the spread of PEDV. In doing so, we have guarded against identifying a too large number of predictors by controlling for sampling bias. We find strong support for live swine trade as a driver of the local and global PEDV G2 epidemic. However, we acknowledge that trade data can be variable through time and is challenging to obtain. In the case of the Chinese live swine trade data, supplementary figure S5, Supplementary Material online, shows a steady year-on-year increase between 2017 and 2018 (no data for the trade of live swine between China’s provinces are available prior to 2017). However, due to the impact of ASFV on the trade of live swine within China and the measures imposed by the MARA, this is not the case for 2019 (as can be seen in supplementary fig. S4, Supplementary Material online). Live swine trade information was missing for many pairs of countries in our global analysis, as can be seen in supplementary figure S10, Supplementary Material online. It is not always clear if this is meant to imply that there is no trade between a given pair of countries, or if these data are simply missing. This is due to the fact that Comtrade combines multiple data sources and the sporadic nature of the numbers being reported (sometimes only one country reports a trade flow). This presents a possible limitation in our study, as any missing trade information is treated by the analysis as a (near) zero value. When a trade flow was reported unilaterally, we opted to include the data that indicated presence of trade over using a missing value. We cross referenced the obtained values with other sources of information but could not find any indications of inconsistency that would potentially alter our inference results. Further, live swine trade data and the trade of related swine products have been subject to drastic changes over the past years. Per capita meat consumption has increased as a result of rising income in many Asian countries and fueled the (global) trade of live swine. For example, at the end of 2017, Singapore started importing its first shipments of live swine from Malaysia in 18 years. In 2016, 767,375 pigs were imported from Vietnam into China, a number that decreased to 47,304 in 2017 and to only 625 in 2018. A final example is Belgium, where as a consequence of ASFV, export of swine and swine products to 24 countries outside of Europa—including to China, India, Vietnam, Singapore, and South Korea—was prohibited until the end of 2019. The variable nature of these trade data presents a particular challenge for modeling the contribution of live swine trade to the local (i.e., within China) and global spread of PEDV. A possible extension of the GLM we used could be to employ an epoch structure (Bielejec et al. 2014) in case trend changes through time could be properly delineated or identified. This would give rise to a multiepoch phylogeographic model which would create a number of discrete diffusion processes to infer spatiotemporal history. Although more plausible to model the evolution of live swine trade over time, the use of such an epoch-GLM has computational and potentially statistical ramifications given the added complexity and increase in the number of parameters, as the number of sequences to inform each epoch interval would be limited.

Few studies have performed detailed investigations into the time and place of origin of the PEDV G2 epidemic. We have estimated mean evolutionary rates of 1.93E-3 substitutions per site per year (95% HPD: [1.83E-3; 2.03E-3]) for the global analysis and 1.23E-3 substitutions per site per year (95% HPD: [1.11E-3; 1.35E-3]) for the within-China analysis, with corresponding tMRCAs of December 2001 (95% HPD: [April 1998; December 2004]) for the global analysis and April 2002 (95% HPD: [April 2000; February 2004]) for the within-China analysis. Earlier studies have found diverging, but roughly similar results concerning the evolutionary rates for the spike protein in PEDV. For example, Stott et al. (2017) found evolutionary rates between 1E-3 and 7.5E-3 substitutions per site per year, (Lee et al. 2019) found evolutionary rates of 14.80E-4 and 7.18E-4 substitutions per site per year (depending on geographic location), and (Jarvis et al. 2016) reported an evolutionary rate of 1.50E-3 substitutions per site per year. The estimated time of origin predates the first known cases of PEDV G2 in 2010 by several years. One previous study reported a tMRCA for G2 PEDV of 1988, that is, 14 years earlier than our own estimate (Jang et al. 2018). However, note that the authors included both G1 and G2 sequences in their analysis, hence the difference in temporal signal and evolutionary rate dynamics between the G1 and G2 clades could explain this discrepancy. Note that the earliest G2 sequence identified in our data set (i.e., *n* after the G1/G2 detection and the recombination analyses) was a Chinese sequence dated to October 16, 2006. This sequence was not included in our final data set as a result of the TempEst analysis, but its existence further suggests the existence of PEDV G2 for quite some years before 2010. Why G2 variants did not cause any noticeable outbreaks, nor produce a fair number of sequences before 2010 has been a topic of discussion for years. We can speculate that G2 has become more infectious over time, leading to cryptic transmission before 2010. However, it does seem that there was sufficient divergence to explain possible phenotypic differences (see the figure below of a sample of G2 sequences from China including the oldest ones; note that there are clear issues with the top 2007 sequence, which is one of the sequences that was removed from our data set after a TempEst analysis). The functional host cell receptor for PEDV remains unidentified (Zhao et al. 2021), yet PEDV has been shown to infect and replicate in porcine, simian, and human cells (Wrapp and McLellan 2019) and these abilities may have changed/improved over time. Identification of the PEDV key receptor in the future may assist in identifying additional G2 sequences to fill the gaps in our data collection. Adding to this point is the fact that early G2 outbreaks may have remained overlooked due to a lack of routine PCR detection efforts before 2010. The current availability of next-generation sequencing however may enable sequencing old PEDV samples. These combined aspects offer the possibility of studying the early history and more accurately pinpoint the inferred time of origin of both the within-China and global PEDV epidemic.

There have been a few studies who have estimated the origin of the American PEDV G2 epidemic, which place the tMRCA around 2010, corresponding to our own results (Huang et al. 2013; Jarvis et al. 2016). This would pre-date the discovery of PEDV G2 in the United States in 2013 by 3 years. There are thus indications that PEDV G2 might have circulated undetected for a significant amount of time. Although it is not unusual to obtain a tMRCA preceding the discovery time of a virus by several years (Smith et al. 2009; Nelson et al. 2011; Michaud et al. 2013; Lo Presti et al. 2016), the 95% HPD interval for the tMRCA in the global data set reflects considerable uncertainty, which can in part be attributed to the use of an uncorrelated relaxed clock model. Including a wider range of dates and increasing the overall number of sequences may help narrow down the uncertainty in the tMRCA estimation. Note that for some locations, such as the United States, the range of sampling dates is quite narrow, with all but 18 of the 384 American sequences being collected between 2013 and 2014. To test the impact of this abundance of American samples during this narrow time period, we reran our global analysis using two subsampling schemes, one with only half the sequences from the United States and one with half the sequences from both the United States and China. The estimated Markov jump plots showed little difference compared with the analyses on the full data set, as can be seen in supplementary figure S16, Supplementary Material online. In terms of the GLM analyses, the results are similar besides the fact that one more variable is found to be significant (import at destination). Regarding the geographic origin of the global PEDV epidemic, previous work has suggested that the first introduction of PEDV into China occurred via South Korea, possibly in connection with a recombination event (Lee 2015). This hypothesis is based on the discovery of early South Korean samples (CHINJU99 and strains from Iksan, South Korea in 2002 and 2009), which may constitute among the earliest reported G2 sequences (Yeo et al. 2003; Kang et al. 2005; Lee et al. 2010; Sun et al. 2015). These sequences, however, were excluded in our analysis during the recombination analysis. Although our data set enables us to infer the timing and origin of local transmission within China, without older unrecombination sequences we remain unable to look more closely at the origin of the G2 clade in Asia. Running our entire analysis pipeline utilizing complete genomes also constitutes an interesting avenue to further uncover the early history of the G2 epidemic (Dudas and Bedford 2019), provided that recombination issues can be properly dealt with.

Finally, we have focused our study on the global dynamics of PEDV in swine, but our findings invite investigation of how trade, quarantine, and swine-farming practices affect the spatial dynamics of other globally dispersed swine pathogens, such as PRRSV and ASFV, or other emerging virus spread among livestock in the future. Modeling studies rooted in pathogen sequence information, demographics, and trade data have the power to inform global surveillance and control strategies for major animal-origin infectious disease threats.

## Materials and Methods

### Sequence Data

As part of a previous nationwide swine virome metagenomics research project (He et al. 2020), we had collected PEDV sequences from 21 Chinese provinces during 2017–2020. In PEDV, the *S1* gene carries important biological functions with a rapid rate of evolution and is often used as a genetic marker to differentiate PEDV genotypes with the largest number of reference sequences (Li et al. 2016). Given these characteristics, the *S1* gene is often used to characterize PEDV genetic diversity and evolution, and we chose to select only this gene our study (Wen et al. 2018). To obtain the *S1* gene of PEDV, we designed a pair of primers for PCR amplification (forward: ATAACGATGTTACAACAGGTCGT; reverse: TAGCACAATCAACACTAACAGG). PCR products were amplified with 30 cycles at 95 °C for 1 min, 56 °C for 1 min, and 72 °C for 3 min, with a final extension at 72 °C for 10 min and sequenced using Sanger sequencing by Sangon Biotech (Shanghai) Co., Ltd. A total of 247 *S1* genes were sequenced and submitted to the National Center for Biotechnology Information’s GenBank (accession numbers MW330010–MW330256). In addition, we downloaded all available PEDV *S1* sequences on GenBank up until July 2, 2020. Sequences that were found to have/be either: 1) missing sampling dates, 2) duplicate copies where the sampling location and time were an exact match, 3) cell passage sequence, 4) vaccine strain, and 5) low-quality sequences, were removed. Multiple sequence alignments were constructed using MAFFT v7.471 (Katoh and Standley 2013) and manually inspected and corrected where necessary. The final data set consisted of 2,371 sequences, which we used in subsequent analyses.

### Recombination Analysis

Recombination in virus genomes can have a negative impact on the accuracy of phylogenetic reconstruction. Previously, evidence has been found for the presence of recombination in PEDV genomes (Arenas and Posada 2010; Li et al. 2016). We found that the N-terminal region of the *S1* gene was often classified as being recombinant (not shown). In order to reduce the number of sequences marked as recombinant, we decided to remove the 1AA-230AA part of the *S1* gene. We subsequently used RDP4 (Martin et al. 2015), which offers formal testing for recombination using up to eight different methods, to further identify and remove recombinant sequences from our data set. For our analysis, the following methods were considered: RDP (Martin and Rybicki 2000) MAXCHI (Wiuf et al. 2001), CHIMAERA (Arenas and Posada 2010), 3SEQ (Boni et al. 2007), and GENECONV (Padidam et al. 1999). We ran two additional approaches, BootScan (Salminen et al. 1995) and SiScan (Gibbs et al. 2000), as secondary methods. We used the recommended settings for all methods, and sequences that were classified as recombinant by three or more methods were subsequently removed from the data set in an iterative manner until no more sequences were flagged as recombinant. In total, 545 putative recombinant sequences were detected and removed from the data set.

### G1/G2 Clade Detection

We subsequently aimed to assess the temporal signal in the resulting data set of 1,826 sequences. By exploiting the sequence data and associated sampling times, a molecular clock can be calibrated to perform time-stamped phylogenetic inference. The estimated evolutionary rate can vary across substrains of a virus, making it potentially difficult to obtain reliable divergence times. Around 2010, a new variant of PEDV emerged in China, gradually becoming the main subtype worldwide. We can currently classify PEDV into either this newer strain (known as G2) or the historical and more sporadic G1 strain (Lee 2015). PEDV sequences are not easily assigned to either the G1 or G2 clade, even though G1 is known to be a paraphyletic group with G2 nested inside of it. We hence performed a formal classification study to accurately identify the collected PEDV sequences as either G1 or G2. We constructed a maximum-likelihood (ML) phylogeny using IQ-TREE 2 (Nguyen et al. 2015), making use of ultrafast bootstrap (UFBoot) (Hoang et al. 2018) to compute 1,000 bootstrap replicates. We performed multiple independent IQ-TREE 2 analyses with increasingly demanding search settings and selected the tree with the highest ML. We performed midpoint rooting on this tree and resolved any polytomies using the *phytools* and *ape* packages in R (Revell 2012; Paradis and Schliep 2019). We then used this rooted ML tree along with the bootstrap support values to perform an automated ClusterPicker (Ragonnet-Cronin et al. 2013) analysis to identify monophyletic clusters. ClusterPicker works as follows: starting from the root, the tree is divided into subtrees, the sequences within the subtree are identified and their pairwise genetic distances are calculated. If the largest of these is smaller than or equal to the user-provided maximum genetic distance threshold, the group of sequences is identified as a cluster. If the maximum pairwise distance is larger than the provided threshold, the cluster is rejected and the algorithm proceeds to inspect a different subtree. ClusterPicker thus offers a within-cluster versus between-cluster assessment of the genetic distances in the phylogeny. Given that we were interested in the split between G1 and G2, we opted for fairly high settings for bootstrap support and genetic distance. We consistently identified one large clade with 1,745 sequences and four smaller clades together containing 81 sequences. The small clades included the prototype European PEDV strain CV777 (supplementary fig. S1, Supplementary Material online) and were identified as belonging to G1. We tested the impact of various cluster thresholds on the detected clusters but found our results to be robust to these settings. To ensure clear temporal signal in our PEDV G2 data set to estimate time-stamped phylogenetic trees through a molecular clock, we excluded these 81 divergent sequences from our data set.

### Global and Local Data Sets

The aim of this study is 2-fold: studying PEDV on both a local and global scale, by focusing on the spatiotemporal evolution of PEDV within China and across the world. We first constructed a data set to study the evolution and spread of PEDV within China. To this end, we selected the three large clades of PEDV sequences predominantly sampled in China from the ML tree (see supplementary fig. S2, Supplementary Material online; the three large clades are shown in blue). Within these clades, we pruned all non-Chinese clusters—that is, all clades that represented introductions into other countries, even if these clusters end up seeding introductions back into China—from the estimated ML tree. This approach was taken in order to maximize the number of PEDV sequences sampled in China without selecting clades that did not originate within China (i.e., introduction events from other countries into China which may seed new epidemics and would prevent from making this a China-specific analysis). This resulted in a collection of 824 sequences from 26 provinces. TempEst (Rambaut et al. 2016) analysis revealed the presence of 40 outliers that could be linked to very long branches in the ML tree, which we also removed. Finally, we checked if these sequences contained geographic information on the provincial level. When the province of origin was not available, we were able to extract this information either based on the fact that the sequence name contained an ISO 3166-2 code, or by contacting the authors of the publications in which these sequences were made available. For 15 sequences we were however unable to obtain the geographical origin, and we further removed these sequences, resulting in a final data set of 769 sequences from the following provinces: Anhui (45), Beijing (6), Chongqing (3), Fujian (35), Gansu (6), Guangdong (119), Guangxi (16), Guizhou (10), Hebei (20), Heilongjiang (28), Henan (91), Hubei (58), Hunan (14), Inner Mongolia (4), Jiangsu (60), Jiangxi (32), Jilin (15), Liaoning (19), Shaanxi (3), Shandong (46), Shanghai (10), Shanxi (24), Sichuan (72), Xinjiang (1), Yunnan (7), and Zhejiang (25).

For the global data set, we first removed two sequences for which no location information was available. TempEst (Rambaut et al. 2016) analysis revealed the presence of 43 outliers (including the oldest available sequence from China, sampled on October 15, 2006) that could be linked to very long branches in the ML tree, and we further removed these sequences which resulted in a final global data set size of 1,700 sequences. These sequences originated from the following countries: Austria (8), Belgium (1), Canada (6), China (947), Colombia (22), Ecuador (1), France (2), Germany (36), Hungary (2), Italy (5), Japan (87), Mexico (51), The Netherlands (10), The Philippines (1), Romania (2), Slovenia (3), South Korea (103), Thailand (3), Ukraine (1), United States (384), and Vietnam (25). We provide a histogram of the available sequences and those that were removed as a result of the TempEst analysis, in supplementary figure S3, Supplementary Material online. Given the low number of sequences from individual European countries, we aggregated these sequences into a single “European” location consisting of 70 sequences. This aggregation makes for a set of locations similar to those employed in an influential global swine influenza study (Nelson et al. 2015).

### Bayesian Phylogeographic Analysis

We performed a joint estimation of the phylogeny and the dispersal history using BEAST v1.10.4 (Suchard et al. 2018). To reconstruct the spatial dispersal process, we modeled the instantaneous rate of transitions between the different states as a continuous-time Markov chain (CTMC) process (Lemey et al. 2009). Under this formulation, movement between *K* discrete locations (i.e., the 26 provinces in the analysis that focuses on China, and 12 countries for the global analysis) is parameterized in terms of a *K* × *K* infinitesimal rate matrix Λ, where Λ_*ij*_ is the instantaneous, relative transition rate from location I to j. We parameterized these rates as a function of a number *P* of potential explanatory predictors in a GLM framework (Lemey et al. 2014). The relative transition rates Λ_*ij*_ are defined as a log linear function of these *P* predictors, with each predictor having a coefficient β_*p*_ for *p* = 1, …, *P* that quantifies its contribution to Λ, and an indicator variable δ_*p*_ that determines its inclusion or exclusion in the model. We employed Bayesian stochastic search variable selection (BSSVS) to explore the space of 2^P^ possible predictor combinations within the GLM, and to obtain a posterior probability on the indicator variables δ_*p*_ which enables assessing each predictor’s relative contribution to PEDV spatial spread. We assigned Bernoulli prior probability distributions on δ_*p*_, to ensure that 50% of the prior probability mass is placed on no predictor being included. We assume a priori that all β_*p*_ were independent and normally distributed, with mean 0 and standard deviation equal to 2 (Lemey et al. 2014). This approach allows for a potentially large number of predictors being evaluated simultaneously (Hong et al. 2020). To determine the degree of support for each predictor, we make use of the Bayes factor cut-offs proposed by Kass and Raftery (1995): a Bayes factor higher than 150 indicates very strong support, a Bayes factor higher than 20 indicates strong support, and a Bayes factor higher than 3 indicates positive support for a predictor’s contribution to the spread of PEDV. We also estimated the expected number of transitions, known as Markov jumps, between each pair of provinces along the phylogenetic branches of the trees (Minin and Suchard 2008a, 2008b).

We describe a total of 17 potential predictors for the phylogeographic reconstruction of PEDV within China (see Supplementary Material online for more details). For province-specific measures (e.g., human population size), we specified an origin and destination predictor that uses the measure at the origin and destination, respectively, as predictor value for each pairwise transition rate, bringing the total number of predictors to 34. Included in this list of predictors are the sample sizes in the different provinces reflecting a considerable heterogeneity among provinces. Importantly, we control for sampling bias, this time by including the residuals for the regression of sample size against case count as in Dudas et al. (2017). These residuals represent how much the actual sample size in a location varies from the expected sample size given the number of cases in that location. If there is sampling bias, we could expect the size of the residuals to influence the transmission dynamics. Of key importance in this study is the availability and assessment of the impact of live swine trade between provinces. Such data are available for the years 2017, 2018, and 2019 (see supplementary fig. S4, Supplementary Material online), with the latter year showing great discrepancies compared with the former two, which can be attributed to the emergence of African swine fever (ASFV) in China in the summer of 2018. First reported in northeastern China in August 2018, ASFV is a highly contagious and often fatal swine disease that quickly spread through the country (Zhou et al. 2018). ASFV caused massive swine deaths in swine farms when imported from abroad, and many long-distance dispatches of live swine were affected and even halted. The monthly live swine trade data show a marked decrease in the number of traded pigs after August 2018 (supplementary fig. S5, Supplementary Material online). Given the impact of ASFV on the trade of live swine from the summer of 2018, we have performed our phylogeographic reconstructions and GLM modeling using live swine trade data from 2017. We added pseudocounts for predictors that had zero entries and subsequently performed log-transformation and standardization on the predictor data. For a detailed description of each predictor, we refer to the Supplementary Material online. We used BEAST v1.10.4 (Suchard et al. 2018) to perform this joint phylogeographic reconstruction, making use of BEAGLE v3.1.0 (Ayres et al. 2019) to improve computational performance on graphic cards for scientific computing.

To reconstruct the global spread of PEDV, we sidestepped the joint estimation of phylogeny and geographic history by fitting the phylogeographic diffusion model to pre-estimated posterior tree distributions. We hence first inferred an empirical distribution of time-calibrated phylogenies using BEAST v1.10.4 (Suchard et al. 2018) with BEAGLE v3.1.0 (Ayres et al. 2019) and subsequently conditioned our phylogeographic reconstructions on an empirical tree distribution of 1,000 phylogenetic trees evenly spaced throughout the posterior simulation after burn-in. We describe a total of eight potential predictors for the global phylogeographic reconstruction of PEDV. For country-specific measures (e.g., human population size), we again specified an origin and destination predictor that uses the measure at the origin and destination, respectively, as predictor value for each pairwise transition rate, bringing the total number of predictors to 14. We again added pseudocounts for null values, standardized, and log-transformed all the variables. We also check for sampling bias. However, we were not able to obtain case counts for the countries in our global data set, making it impossible to run a regression analysis of sample size against case counts. Instead, as in Lemey et al. (2014), we included the sample size by itself as a variable. As can be seen in supplementary figure S11, Supplementary Material online, sample size is highly correlated with some of the other predictors, especially population size, feed production, and gross domestic product (GDP). Our aim here was not to demonstrate a role for sample sizes in the global spread of PEDV, but to raise the credibility that other predictors are not included in the model because of sampling bias by explicitly including them as predictive variables. For a detailed description of each predictor, we refer to the Supplementary Material online. We estimated the Markov jumps between each pair of countries along the phylogenetic branches of the empirical tree distribution (Minin and Suchard 2008a, 2008b).

We made use of the following models in the BEAST analyses that were performed for both data sets. We modeled molecular evolution according to an HKY + Γ4 (Hasegawa et al. 1985; Yang 1994) substitution model with estimated nucleotide frequencies and employing a nonparametric coalescent model known as the skygrid (Gill et al. 2013) as the tree-generative process. Efficient estimation of the skygrid model parameters was achieved using a Hamiltonian Monte Carlo transition kernel (Baele et al. 2020). We employed the default prior distributions as offered by BEAUti. Initial data set explorations revealed that a strict clock was the most appropriate molecular clock model for the within-China analysis, as the posterior estimates of the standard deviation of the popular uncorrelated relaxed clock model with an underlying lognormal distribution included zero. For the global analysis on the other hand, we opted for an uncorrelated relaxed clock with an underlying lognormal distribution. We assumed a CTMC reference prior on the (mean) clock rate parameter (Ferreira and Suchard 2008). A sizeable number of sequences only had partial sampling date information in the form of the sampling month or year. For these sequences, we performed tip-date sampling from a uniform prior that spans the entire month or year. The Markov chain Monte Carlo analyses in BEAST were run sufficiently long to ensure adequate statistical mixing on all relevant parameters as assessed through Tracer 1.7 (Rambaut et al. 2018). For the data set focusing on China, this implied running a total of 2 billion iterations in BEAST for the joint phylogeographic reconstruction, whereas the phylogenetic reconstruction to determine the empirical tree distribution for the global data set required a total of 800 million iterations to achieve adequate statistical mixing. The geographic reconstruction on the empirical trees for the global data set was run for 30 million iterations. We computed maximum clade credibility (MCC) trees for both data sets using TreeAnnotator. Tree visualizations were constructed using FigTree (http://tree.bio.ed.ac.uk/software/figtree/), and visualizations of PEDV on geographic maps were performed using SpreaD3 (Bielejec et al. 2016).

## Supplementary Material

Supplementary data are available at *Molecular Biology and Evolution* online.

## Supplementary Material

msab364_Supplementary_Data
