# Supplementary material for: Phylogeography Reveals Association between Swine Trade and the Spread of Porcine Epidemic Diarrhea Virus in China and across the World
Source: Mol Biol Evol. 2021 Dec 24;39(2):msab364. doi: 10.1093/molbev/msab364 (PMC8826572; doi:10.1093/molbev/msab364)
Supplement: msab364_Supplementary_Data [file msab364_supplementary_data.zip › FigureS1-16.pdf]

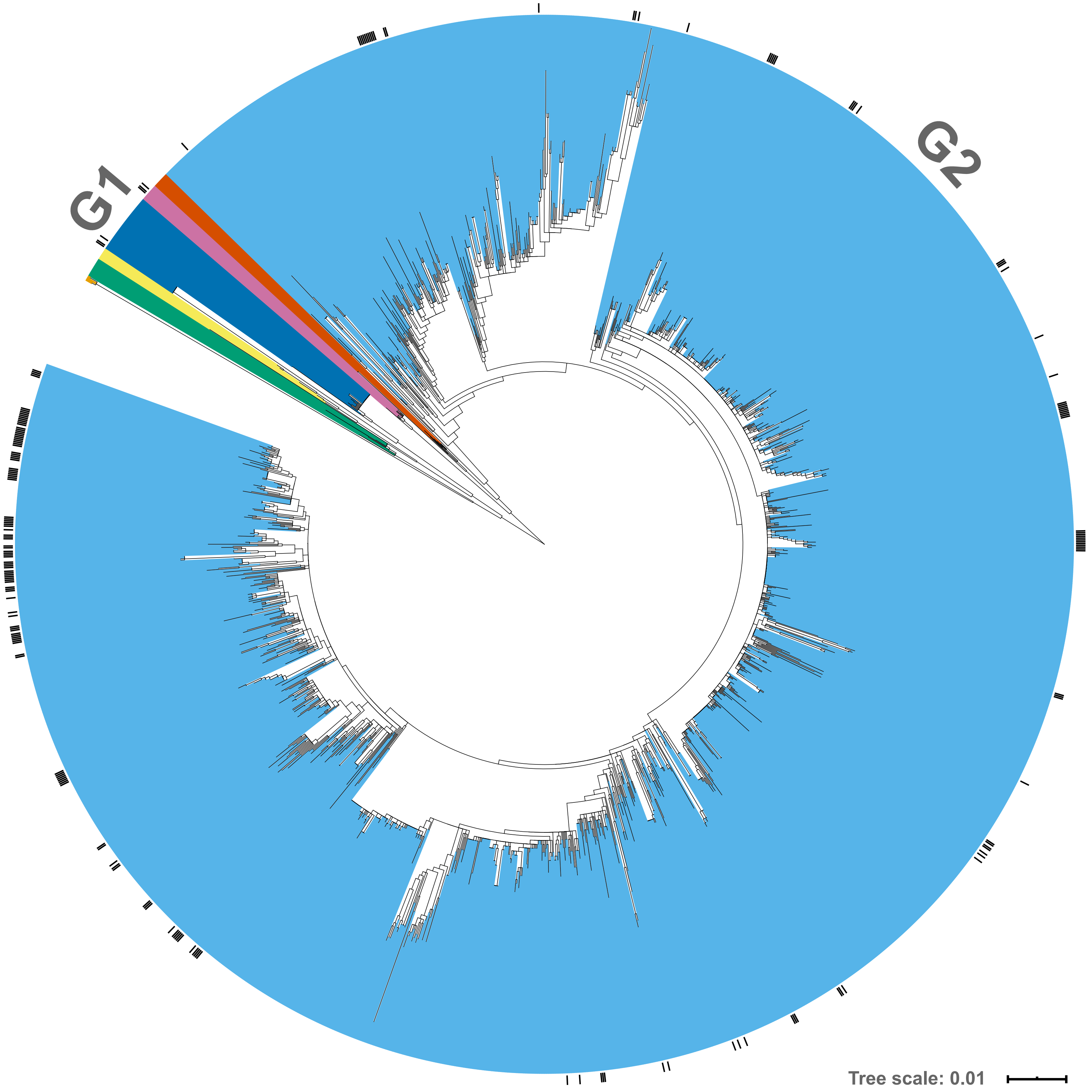
G1
G2
Tree scale: 0.01

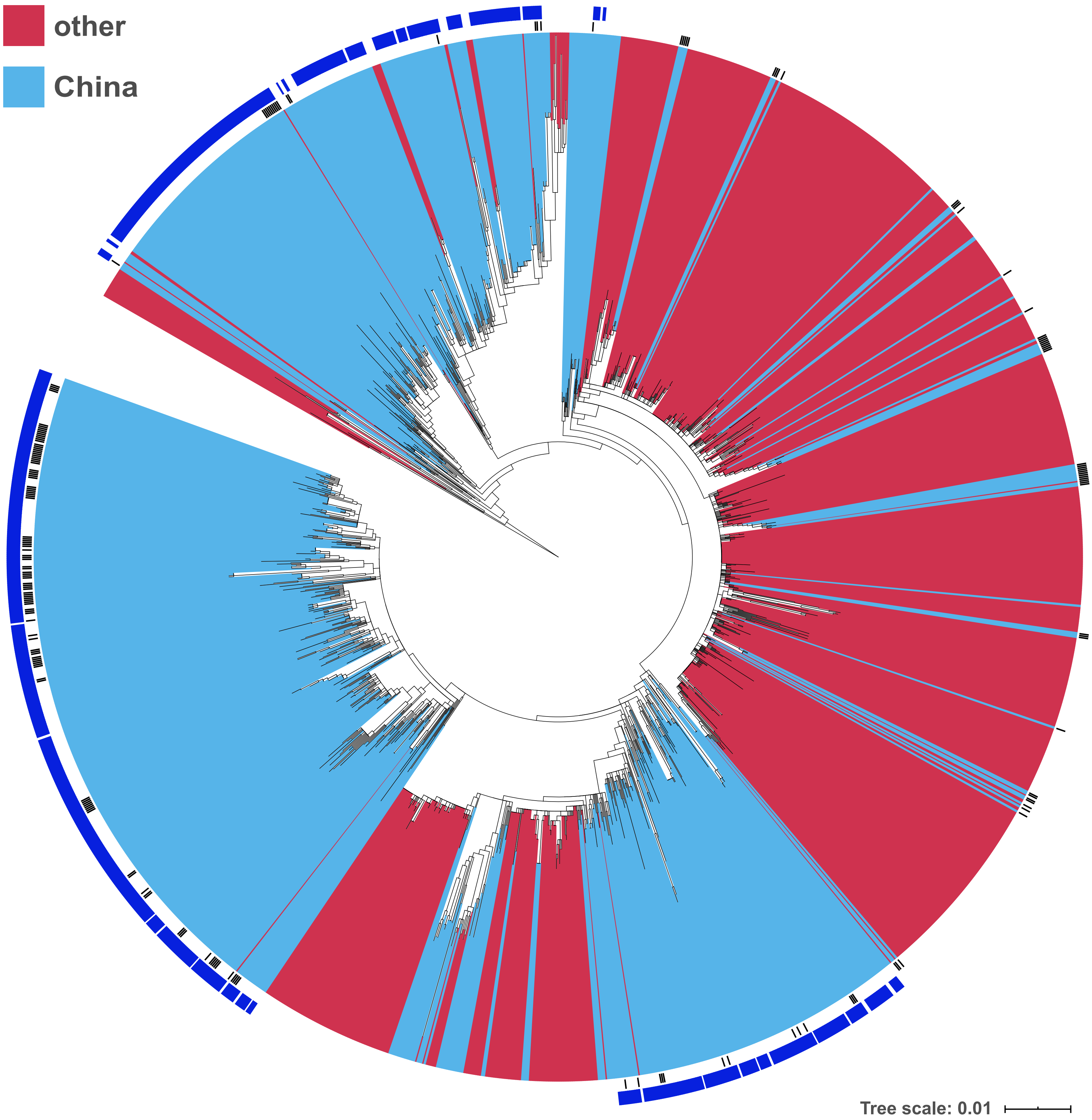
other
China
Tree scale: 0.01

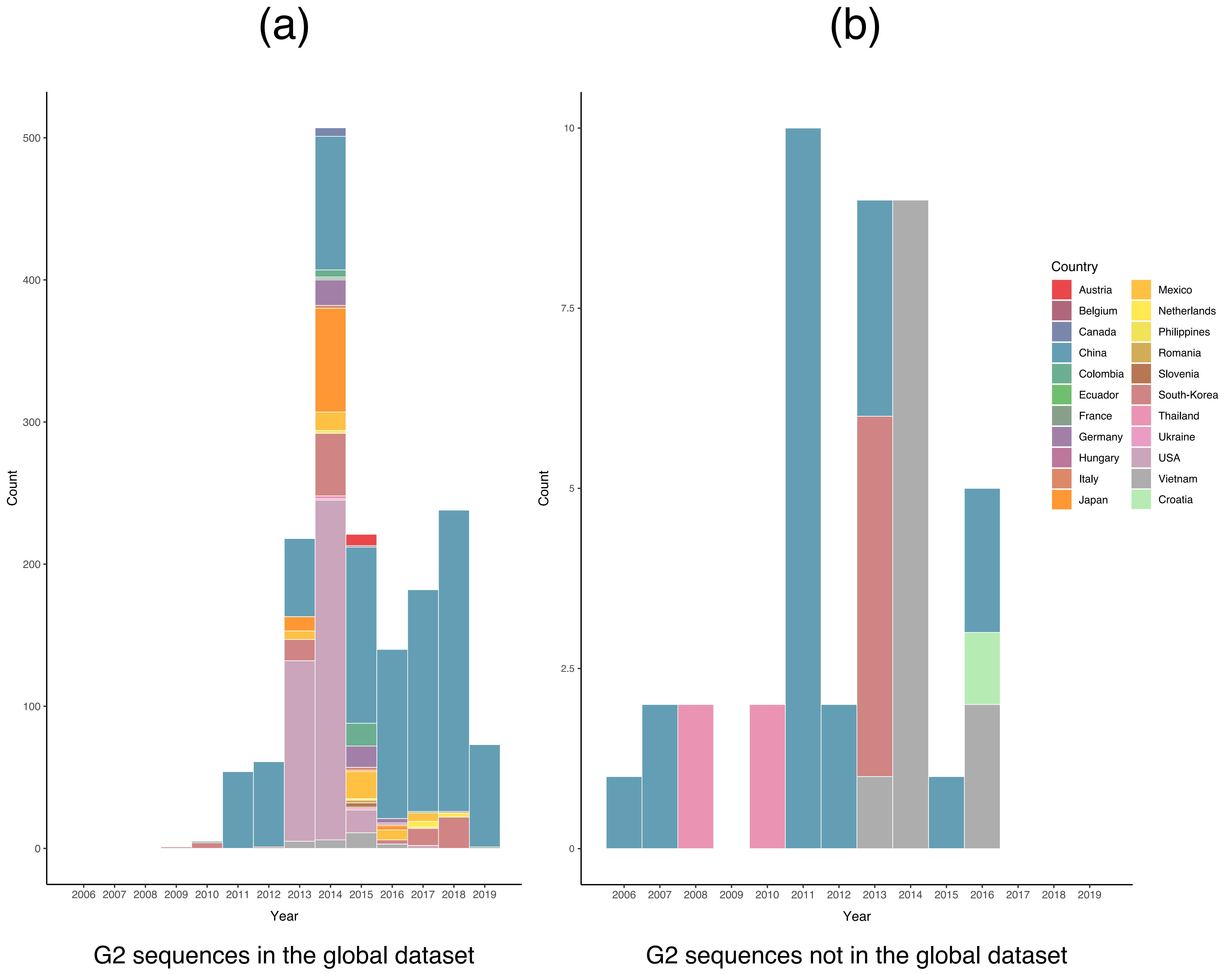
(a)
(b)
Count
Year
2006
2007
2008
2009
2010
2011
2012
2013
2014
2015
2016
2017
2018
2019
0
100
200
300
400
500
2.5
5
7.5
10
Country
Austria
Belgium
Canada
China
Colombia
Ecuador
France
Germany
Hungary
Italy
Japan
Mexico
Netherlands
Philippines
Romania
Slovenia
South-Korea
Thailand
Ukraine
USA
Vietnam
Croatia
G2 sequences in the global dataset
G2 sequences not in the global dataset

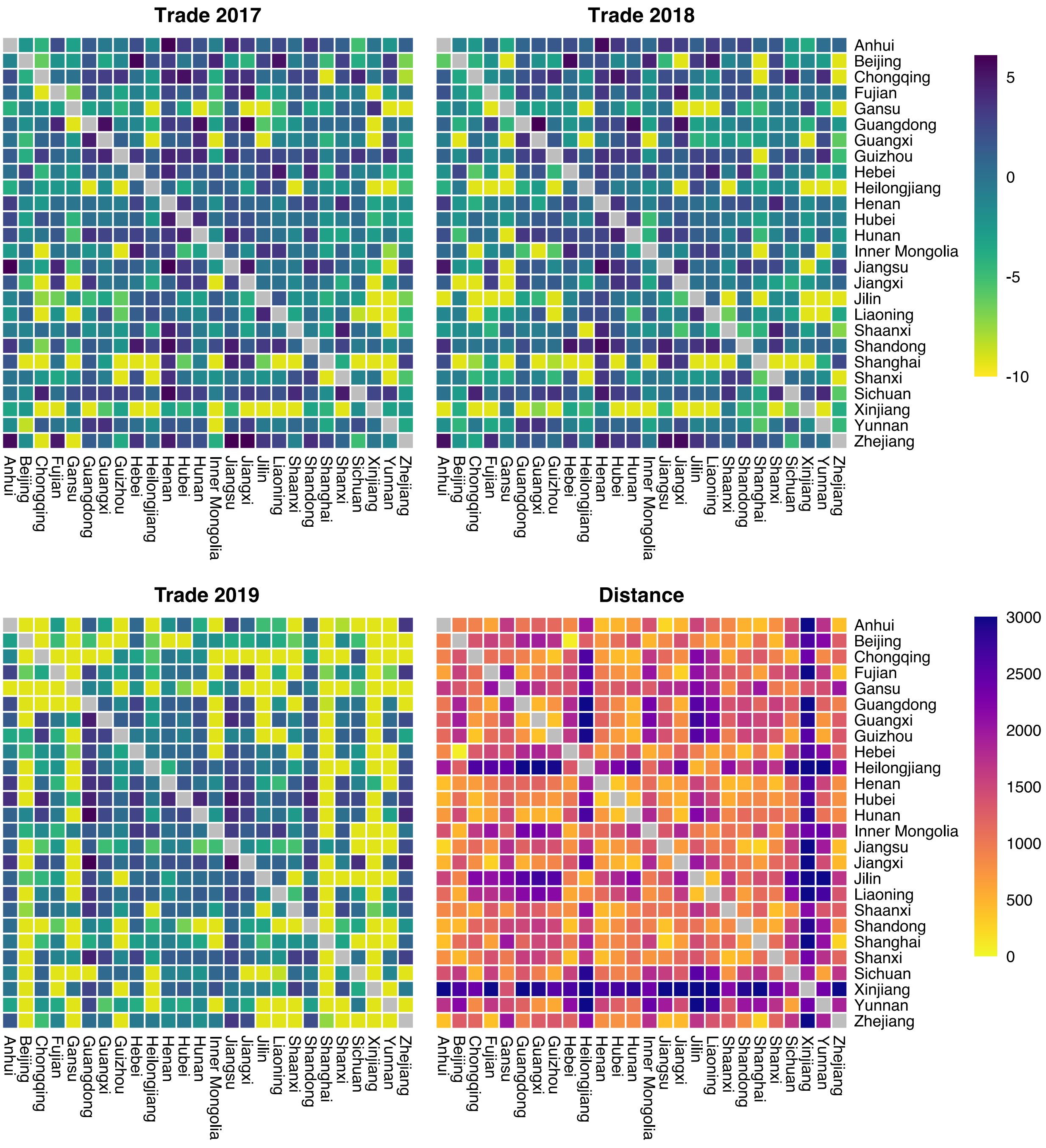

Trade 2017
Trade 2018
Trade 2019
Distance
Anhui
Beijing
Chongqing
Fujian
Gansu
Guangdong
Guangxi
Guizhou
Hebei
Heilongjiang
Henan
Hubei
Hunan
Inner Mongolia
Jiangsu
Jiangxi
Jilin
Liaoning
Shaanxi
Shandong
Shanghai
Shanxi
Sichuan
Xinjiang
Yunnan
Zhejiang
5
0
-5
-10
3000
2500
2000
1500
1000
500
0

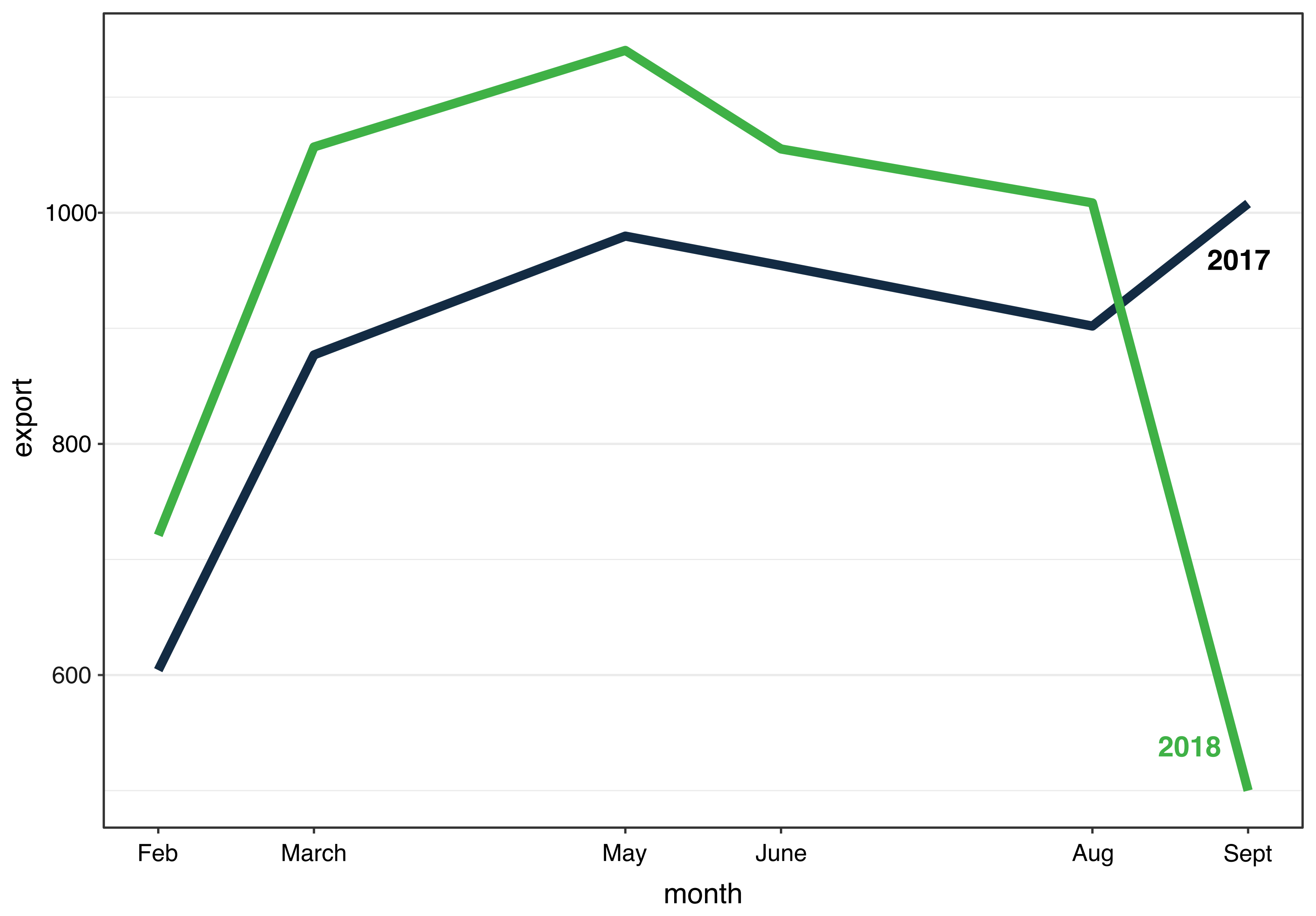

export
1000
800
600
2017
2018
Feb
March
May
June
Aug
Sept
month

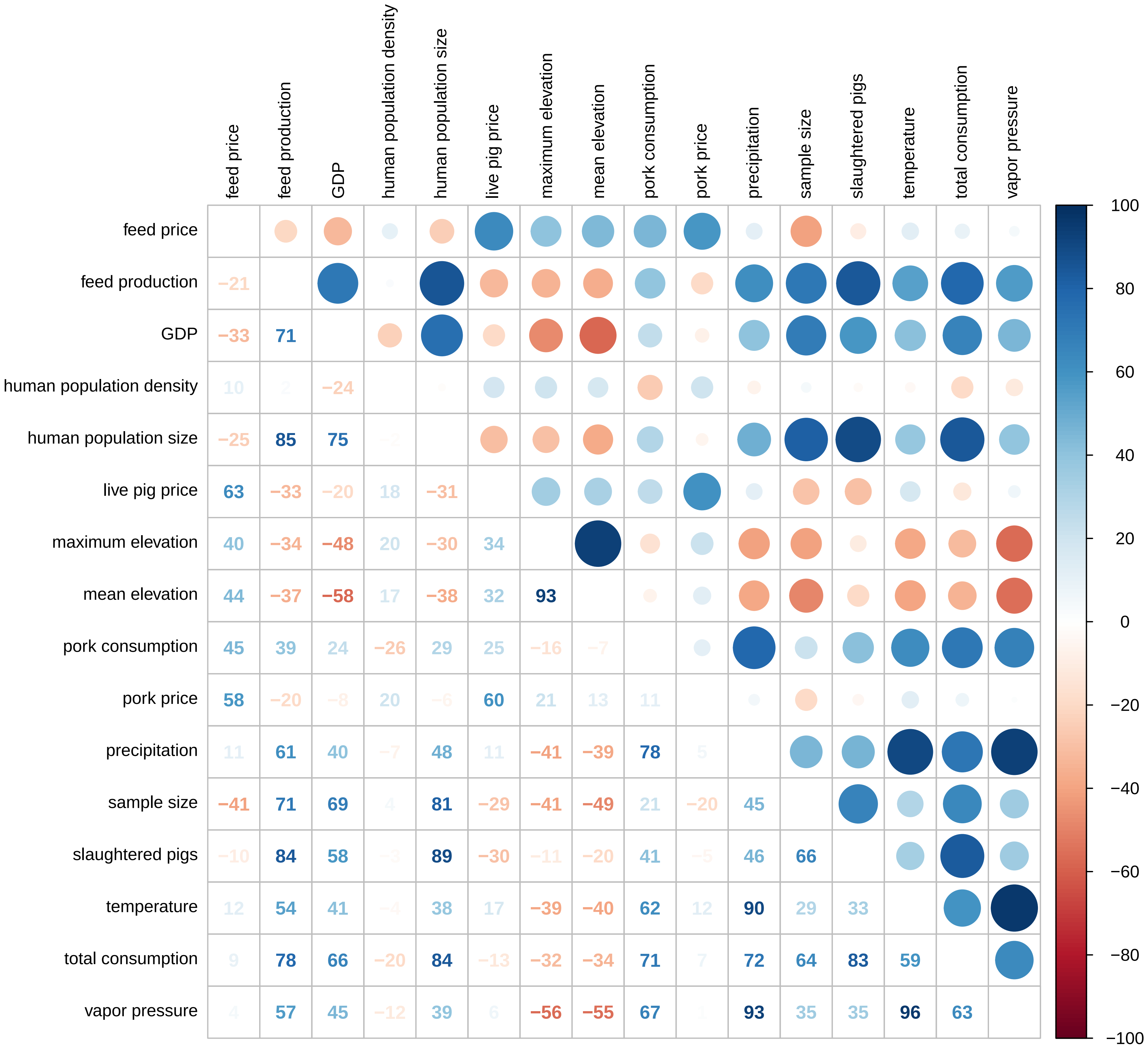

feed price
feed production
GDP
human population density
human population size
live pig price
maximum elevation
mean elevation
pork consumption
pork price
precipitation
sample size
slaughtered pigs
temperature
total consumption
vapor pressure
feed production −21
GDP −33 71
human population density 10 2 −24
human population size −25 85 75 −2
live pig price 63 −33 −20 18 −31
maximum elevation 40 −34 −48 20 −30 34
mean elevation 44 −37 −58 17 −38 32 93
pork consumption 45 39 24 −26 29 25 −16 −7
pork price 58 −20 −8 20 −6 60 21 13 11
precipitation 11 61 40 −7 48 11 −41 −39 78 5
sample size −41 71 69 4 81 −29 −41 −49 21 −20 45
slaughtered pigs −10 84 58 −3 89 −30 −11 −20 41 −5 46 66
temperature 12 54 41 −4 38 17 −39 −40 62 12 90 29 33
total consumption 9 78 66 −20 84 −13 −32 −34 71 7 72 64 83 59
vapor pressure 4 57 45 −12 39 6 −56 −55 67 1 93 35 35 96 63
100
80
60
40
20
0
−20
−40
−60
−80
−100

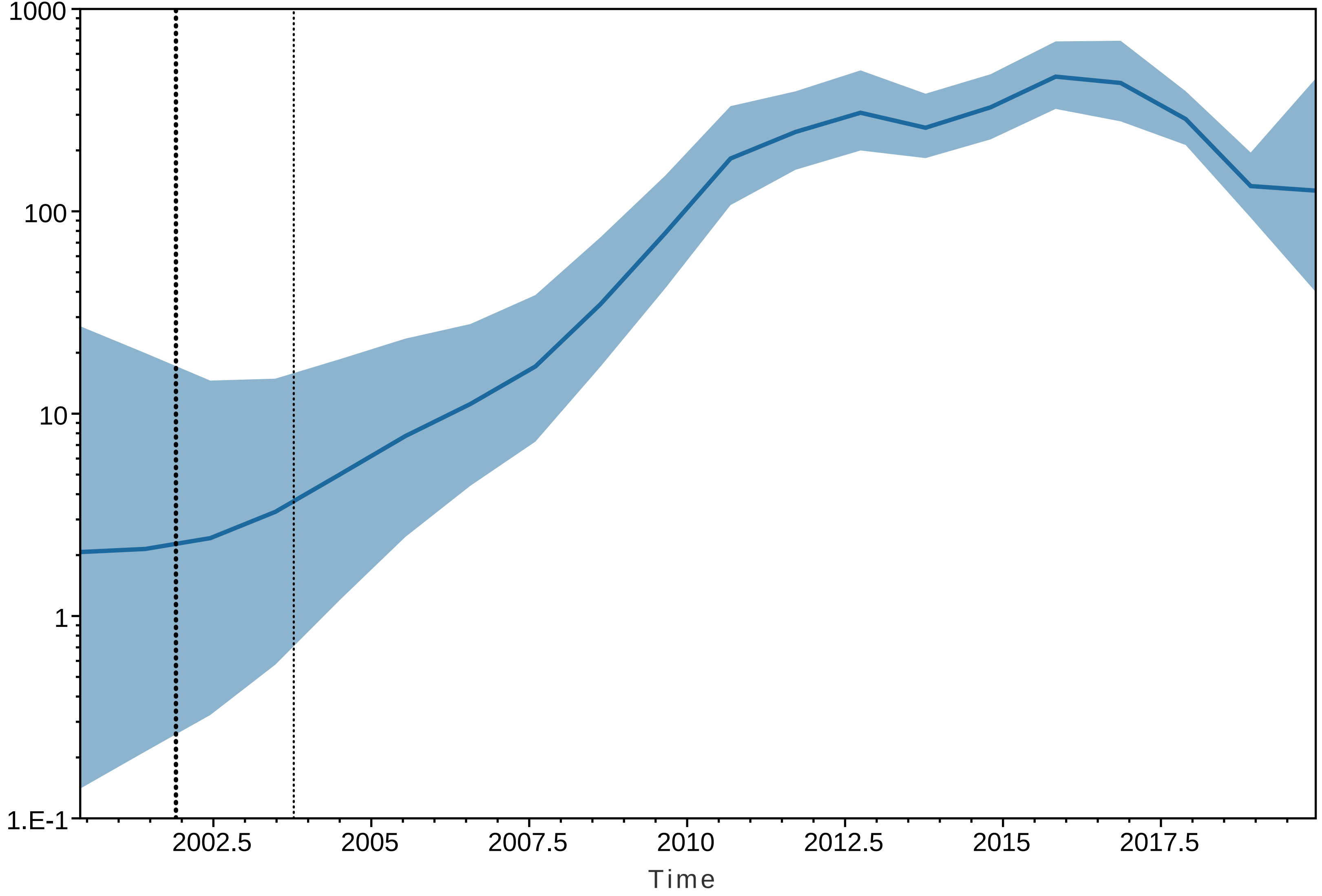

1000
100
10
1
1.E-1
2002.5
2005
2007.5
2010
2012.5
2015
2017.5
Time

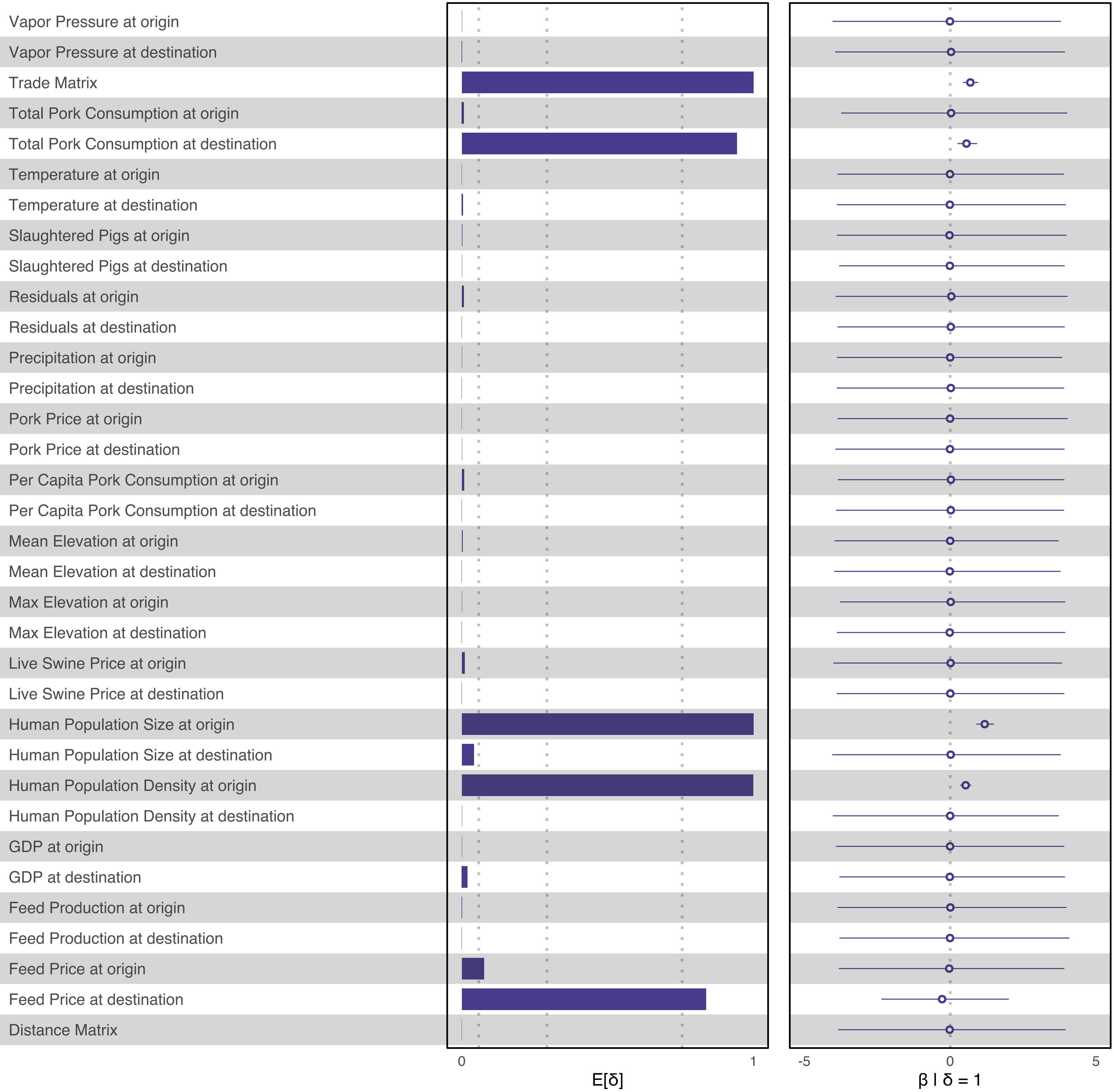

Vapor Pressure at origin
Vapor Pressure at destination
Trade Matrix
Total Pork Consumption at origin
Total Pork Consumption at destination
Temperature at origin
Temperature at destination
Slaughtered Pigs at origin
Slaughtered Pigs at destination
Residuals at origin
Residuals at destination
Precipitation at origin
Precipitation at destination
Pork Price at origin
Pork Price at destination
Per Capita Pork Consumption at origin
Per Capita Pork Consumption at destination
Mean Elevation at origin
Mean Elevation at destination
Max Elevation at origin
Max Elevation at destination
Live Swine Price at origin
Live Swine Price at destination
Human Population Size at origin
Human Population Size at destination
Human Population Density at origin
Human Population Density at destination
GDP at origin
GDP at destination
Feed Production at origin
Feed Production at destination
Feed Price at origin
Feed Price at destination
Distance Matrix
0
1
E[δ]
-5
0
5
β | δ = 1

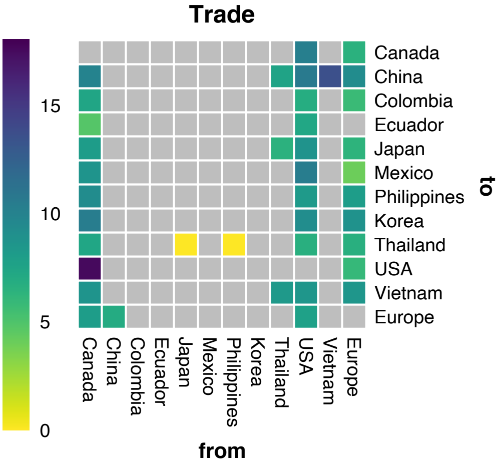

Trade
15
10
5
0
Canada
China
Colombia
Ecuador
Japan
Mexico
Philippines
Korea
Thailand
USA
Vietnam
Europe
to
Canada
China
Colombia
Ecuador
Japan
Mexico
Philippines
Korea
Thailand
USA
Vietnam
Europe
from

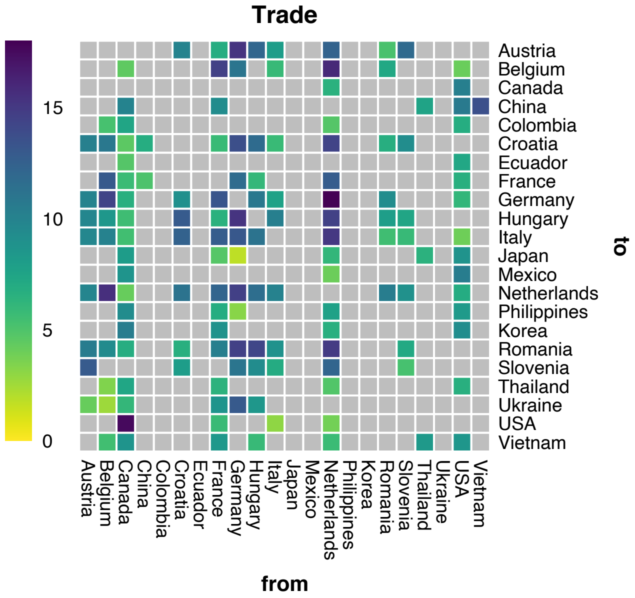

Trade
0
5
10
15
Austria
Belgium
Canada
China
Colombia
Croatia
Ecuador
France
Germany
Hungary
Italy
Japan
Mexico
Netherlands
Philippines
Korea
Romania
Slovenia
Thailand
Ukraine
USA
Vietnam
to
from

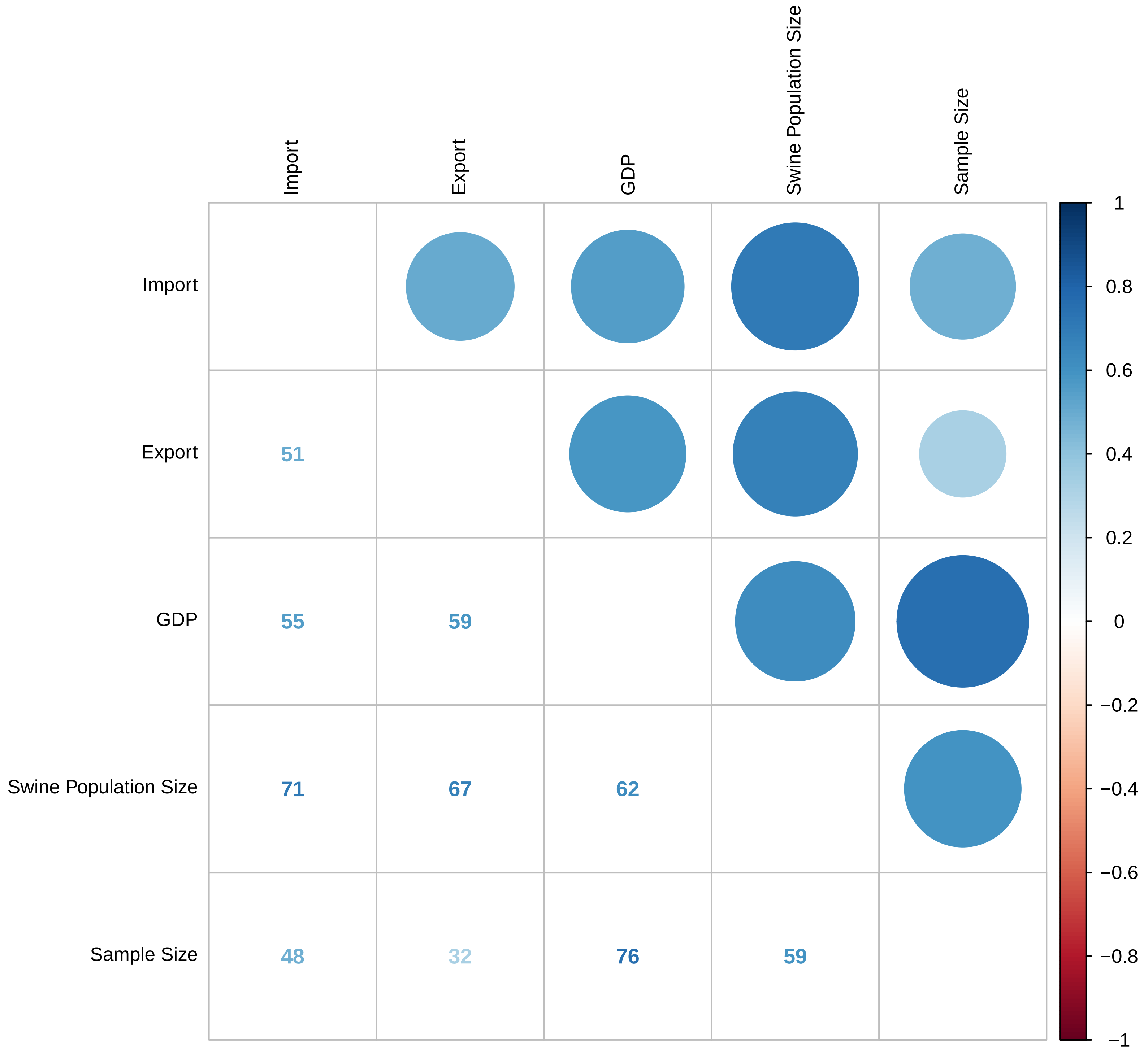
Import
Export
GDP
Swine Population Size
Sample Size
Import
Export
GDP
Swine Population Size
Sample Size
51
55
59
71
67
62
48
32
76
59
1
0.8
0.6
0.4
0.2
0
−0.2
−0.4
−0.6
−0.8
−1

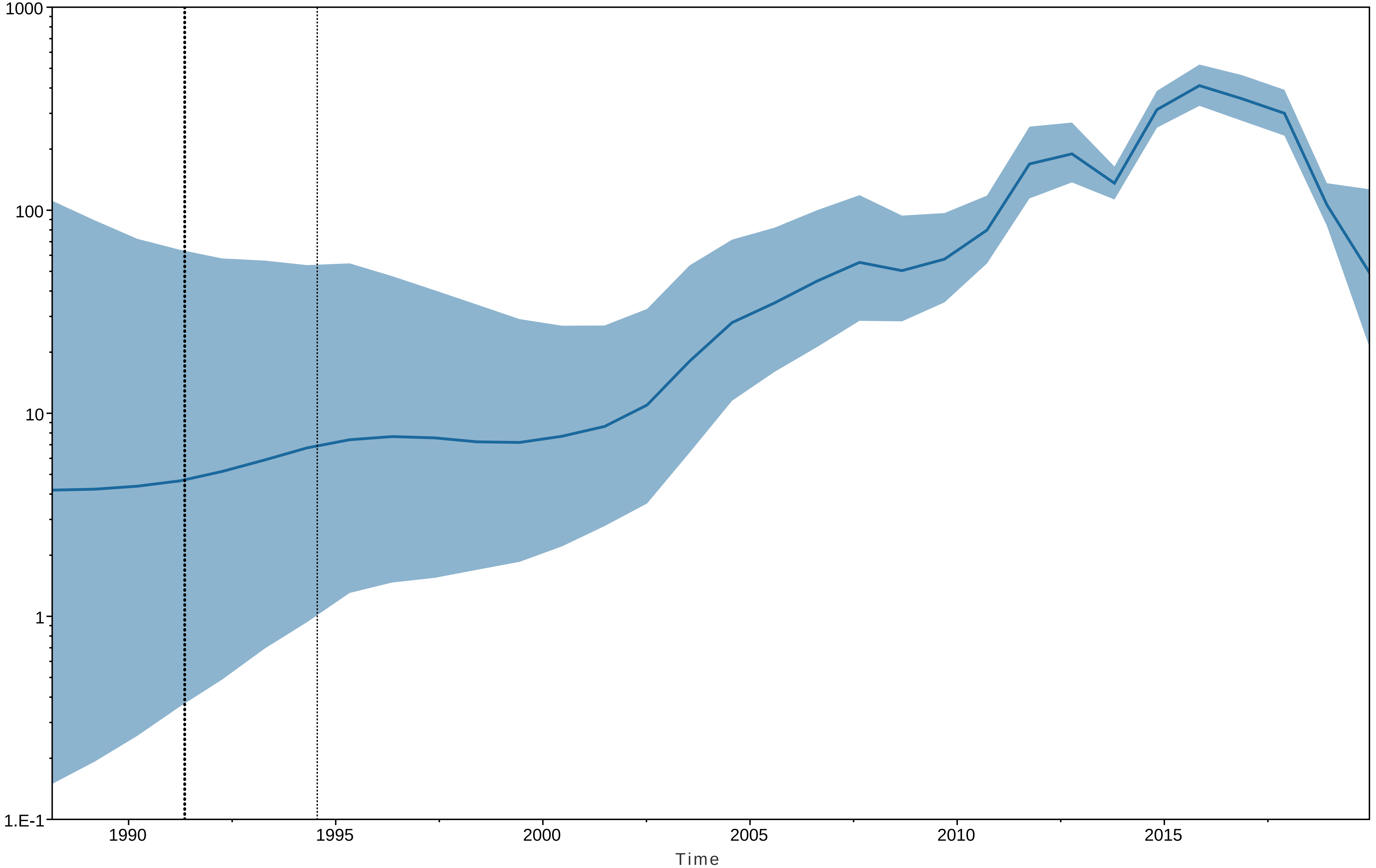

1000
100
10
1
1.E-1
1990
1995
2000
2005
2010
2015
Time

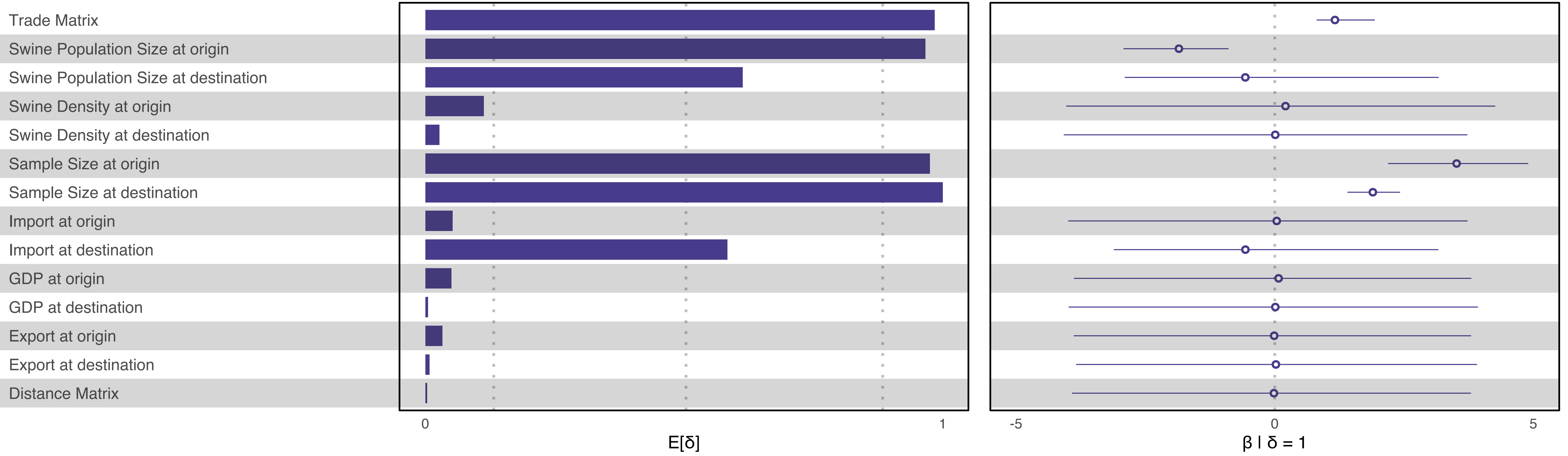

Trade Matrix
Swine Population Size at origin
Swine Population Size at destination
Swine Density at origin
Swine Density at destination
Sample Size at origin
Sample Size at destination
Import at origin
Import at destination
GDP at origin
GDP at destination
Export at origin
Export at destination
Distance Matrix
0
1
E[δ]
-5
0
5
β | δ = 1

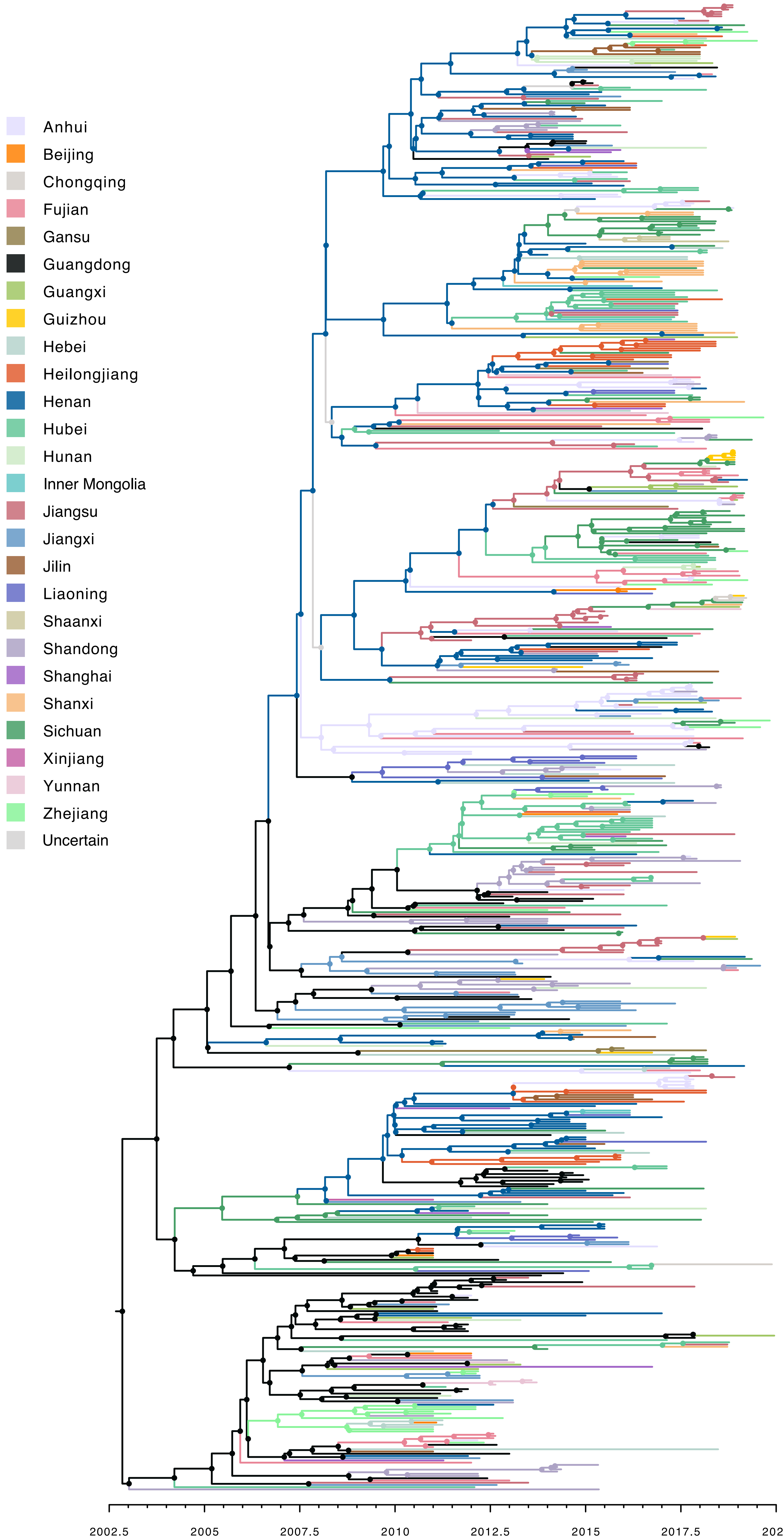
Anhui
Beijing
Chongqing
Fujian
Gansu
Guangdong
Guangxi
Guizhou
Hebei
Heilongjiang
Henan
Hubei
Hunan
Inner Mongolia
Jiangsu
Jiangxi
Jilin
Liaoning
Shaanxi
Shandong
Shanghai
Shanxi
Sichuan
Xinjiang
Yunnan
Zhejiang
Uncertain
2002.5
2005
2007.5
2010
2012.5
2015
2017.5
202

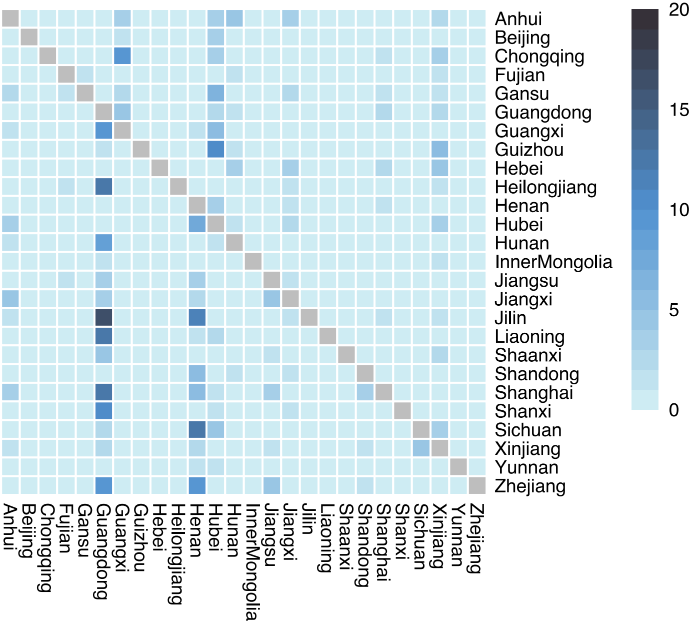

Anhui
Beijing
Chongqing
Fujian
Gansu
Guangdong
Guangxi
Guizhou
Hebei
Heilongjiang
Henan
Hubei
Hunan
InnerMongolia
Jiangsu
Jiangxi
Jilin
Liaoning
Shaanxi
Shandong
Shanghai
Shanxi
Sichuan
Xinjiang
Yunnan
Zhejiang
20
15
10
5
0

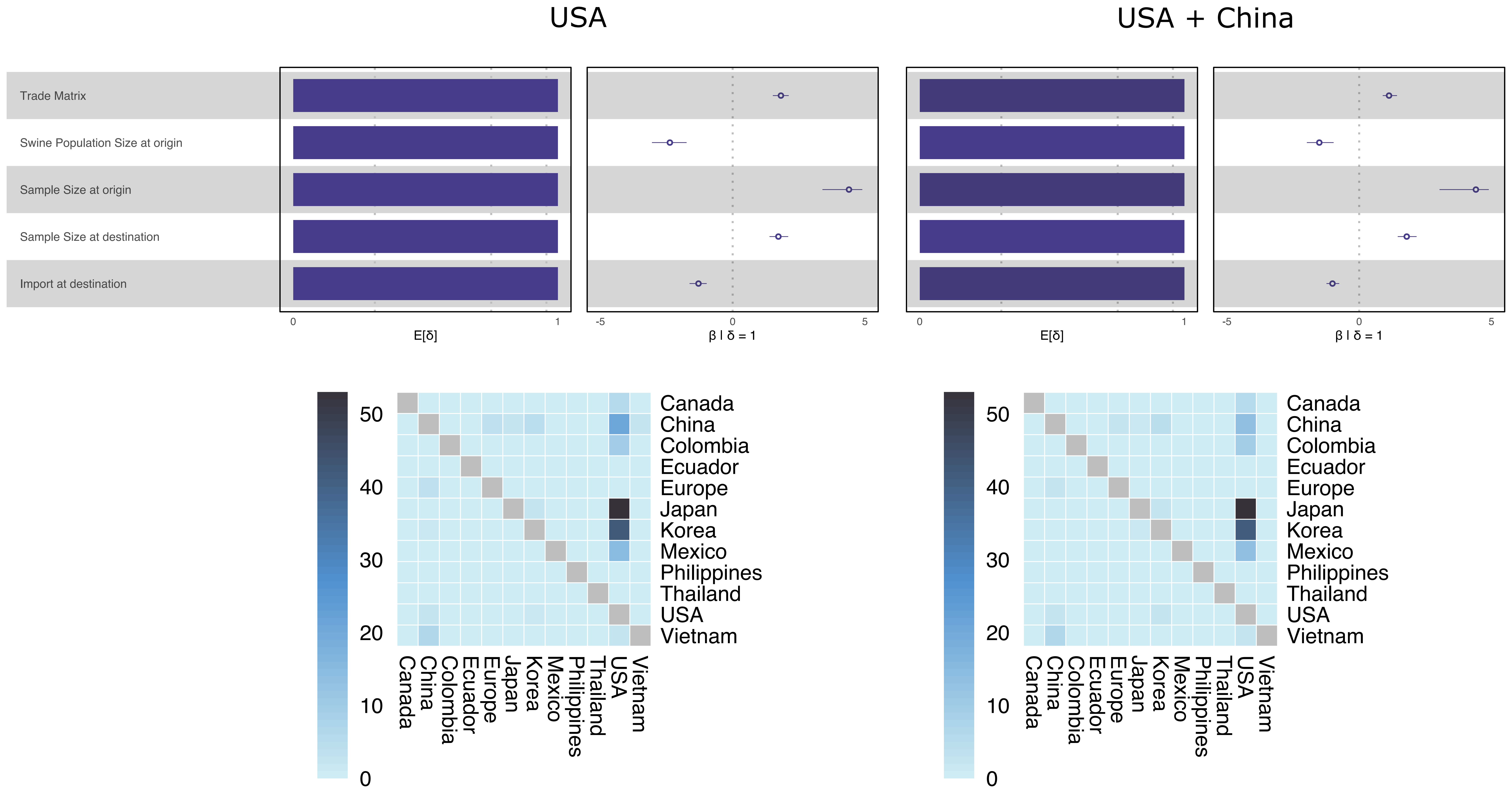
USA
USA + China
Trade Matrix
Swine Population Size at origin
Sample Size at origin
Sample Size at destination
Import at destination
0
1
E[δ]
-5
0
5
β | δ = 1
50
40
30
20
10
0
Canada
China
Colombia
Ecuador
Europe
Japan
Korea
Mexico
Philippines
Thailand
USA
Vietnam
