# Supplementary material for: Phylogeography Reveals Association between Swine Trade and the Spread of Porcine Epidemic Diarrhea Virus in China and across the World
Source: Mol Biol Evol. 2021 Dec 24;39(2):msab364. doi: 10.1093/molbev/msab364 (PMC8826572; doi:10.1093/molbev/msab364)
Supplement: msab364_Supplementary_Data [file msab364_supplementary_data.zip › Table S.pdf]

Table S1. Quantity and amount (in thousands of dollars) of live swine imported into and exported out of China for the period 2009-2019. These data were provided by the National Bureau of Statistics of China (http://www.stats.gov.cn/)

Years	The number of exported live swine (thousand dollars)	Export quantity（Ten thousand tons）	The number of imported live swine (thousand dollars)	Import quantity（Ten thousand tons）
2009	330511	162355	5717	187
2010	338937	168896	7723	253
2011	452778	156458	22020	730
2012	461059	167927	34260	1069
2013	459778	172231	38877	1164
2014	456738	180100	20648	563
2015	483354	178890	8287	262
2016	513640	166748	11199	320
2017	450432	170351	20576	599
2018	427601	N/A	13018	N/A
2019	396409	N/A	3018	N/A

Table S2. Markov Jump Matrix of China.

	Anhui	Beijing	Chongqing	Fujian	Gansu	Guangdong	Guangxi	Guizhou	Hebei	Heilongjiang	Henan	Hubei	Hunan	Inner Mongolia	Jiangsu	Jiangxi	Jilin	Liaoning	Shaanxi	Shandong	Shanghai	Shanxi	Sichuan	Xinjiang	Yunnan	Zhejiang
Anhui	NA	0.2209	0.0004	0.0001	0.0807	0.001	2.0917	0.5236	0.0004	0.5099	0.485	6.2211	1.7412	0.0164	0.0003	2.3384	0.5641	0.0734	0.0628	0.0022	0.7565	0.0222	0.0041	0.829	0	0.0009
Beijing	0	NA	0.0004	0.0017	0.0004	0.0107	0.3242	0.005	0.0158	0.0089	0.1266	6.5952	0.1128	0.5339	0	0.2346	0.3329	0.0139	0.0258	0.0363	0.9728	0.0044	0.0453	0.1331	0.0002	0.0018
Chongqing	0.0196	0.0004	NA	0.0007	0.011	0.0004	7.31	0.0034	0.0023	0.7145	0.0004	7.567	0.3444	0.1933	0.0009	0.1459	0.1233	0.2041	0.092	0.0078	1.2903	0.0001	0.008	2.1366	0.0001	0.0603
Fujian	0.002	0	0.0033	NA	1.0463	0.0003	0.09	0.0394	0.0026	0.0393	0.0335	1.0985	0.6598	0.4386	0.0003	0	0.0015	0.0101	0.0801	0.0619	0.2919	0.0011	0.0006	0.6569	0.0004	0.0066
Gansu	0.6118	0	0.0002	0.0867	NA	0.0007	2.0407	0.0003	0.0864	0.0737	0.9888	13.162	0.0072	0.1344	0.0003	0.9879	0.5656	0.001	0.0105	0.0342	1.2117	0.0006	0.0437	0.4592	0.0001	0.057
Guangdong	0.022	0.001	0	0.0259	0.0017	NA	4.4575	0.2742	0.0008	0.3585	0.0328	4.8712	0.23	0.0056	0.0913	0.0847	0	0.0065	0.0401	0.0105	1.7575	0.0181	0.0144	1.4057	0	0.4112
Guangxi	0.8137	0.0001	0.0029	0.0003	0.0004	6.3672	NA	0.0074	0.0044	0.0091	1.0696	8.9024	0.0255	0.3904	0	0.5828	0.035	0.001	0.0307	0.0029	0.8384	0.0019	0.0819	0.3009	0.0001	0.0023
Guizhou	0.1178	0.0018	0.001	0	0.0002	1.8319	0.0256	NA	0.004	0.0271	0.0005	20.9254	0.1257	0.0722	0.0007	0.049	0.0849	0	0.0081	0.064	0.0493	0.0661	0.0676	4.9802	0.0001	0.0252
Hebei	0.0132	0.0001	0.0079	0.0023	0.0013	0.0923	0.0022	0.007	NA	0.7733	0.0035	0.6016	0.6763	0.0221	0.0002	1.3364	0.005	0.0551	0.6209	0.005	2.8013	0	0.0101	2.4236	0.0012	0.0088
Heilongjiang	0.5097	0.0001	0.0014	0.187	0	11.4767	0.0012	0.0006	0.4908	NA	0.0341	1.9194	0.0029	0.4166	0.0002	1.0831	0.5326	0.0002	0	0.0096	0.7619	0.0322	0.0051	0.4735	0.0006	0.0412
Henan	0.0707	0.0119	0.7885	0.0327	0.0077	0.1447	0.0588	0.0348	1.1606	0.1457	NA	6.8588	0.0276	0.0078	0.0008	0.4257	0.0365	0.0058	0.0008	0.0118	0.9571	0.0051	0.0765	0.0324	0.0003	0.0308
Hubei	1.7536	0.0019	0.0039	0.0017	0.0007	0	0.0052	0.0079	0.0029	0.0771	15.4638	NA	0.1065	0.0514	0	1.2688	0.0079	0.0012	0.5072	0	0.6293	0.0008	0.0074	1.4301	0.0001	0.0156
Hunan	0.4773	0.0011	0.0008	0.066	0.743	7.3913	0.1083	0.0903	0.8031	0.001	1.5294	0.1981	NA	0.443	0	0.0067	0.2792	0.0008	0.0028	0.0141	0.016	0.0004	0.0058	0.2311	0.0003	0.0006
InnerMongolia	0.1244	0.0007	0.0119	0.0089	0.0343	1.0378	0	0.0515	0.2397	0.0166	0.1645	0.0091	0.2312	NA	0.0004	0.0435	0.0011	0.0087	0.0259	0.0045	0	0.0001	0.0119	1.1069	0	0.0288
Jiangsu	0.0122	0.0006	0.0095	0.2741	0.0298	3.0531	0.0193	0.0244	0.3092	0.0639	10.4913	0.9333	0.0318	0.0002	NA	0.3612	0.0203	0	0.0068	0.022	0.0853	0.0052	0.0161	0.2155	0	0.0183
Jiangxi	2.2165	0.0002	0.0034	0.051	0.019	1.259	0.023	0	0.0509	0.0191	4.1141	0.1395	0.0093	0.0001	1.749	NA	0.4432	0.0001	0.0001	0.0578	0.1076	0	0.0312	0.6177	0	0.0069
Jilin	0.6707	0.0007	0.0002	0.0829	0.0018	12.8784	0.0022	0.0252	0.0623	0.0079	16.5939	0.0133	0.3995	0	0.0343	1.1324	NA	0.0081	0.0007	0.0015	1.2682	0.0011	0.0013	0.4157	0	0.0089
Liaoning	0.0719	0.0001	0.0085	0.0011	0.0008	8.3619	0.0494	0.0057	0	0.0008	4.2271	0.3126	0.0369	0.0008	0.0113	0.007	0.054	NA	0.0158	0.0006	0.0176	0.0014	0	0.1101	0.0003	0.001
Shaanxi	0.142	0.0005	0.0037	0.1043	0.0001	5.0813	0.0308	0.0223	0.1656	0.049	1.6998	0.0274	0.5268	0.0001	0.7302	0.0012	0.0008	0.0089	NA	0.0176	0.0578	0.0001	0.5079	2.0742	0.0001	0.0199
Shandong	0.0512	0.0308	0.0014	0.2009	0.0088	0.0424	0.0733	0.0158	0.5627	0	10.8956	0.014	1.7434	0.0008	0.0332	0.9358	0.0001	0.0802	0.0354	NA	0.2312	0.0004	0.0017	0	0	0.0055
Shanghai	2.9674	0	0.0023	0.0072	0.0006	9.2938	0.0004	0.0393	0.3115	0.1456	10.7629	0.3226	0.137	0.0001	1.619	0.0068	0.0013	0.0002	0.003	3.2334	NA	0.0041	0.0014	0.2483	0.001	0.0062
Shanxi	0.0526	0.0021	0.0002	0.0093	0.0169	14.0252	0.0405	0.0006	0.1417	0.0292	0	0.778	0.3677	0	0.2093	1.3985	0.0036	0.0065	0.0011	0.0736	0.0048	NA	0.0121	0.7128	0	0.0666
Sichuan	0.0354	0.0001	0.0058	0.0014	0.0548	0.7584	0.0688	0.0162	0.0372	0.0309	20.238	0.5778	0.4776	0.0021	0.1664	0.1651	0.0039	0.0107	0.0138	0.0032	0.0001	0.0339	NA	2.8843	0.0001	0.0016
Xinjiang	0.2796	0.0002	0.0003	0.1248	0.0008	1.168	0.0019	0.0143	0.2384	0.0168	7.4115	0	0.5775	0.0025	0.7536	0.0715	0.0009	0.0245	0.0097	1.1089	0	0.0032	2.9188	NA	0.0005	0
Yunnan	0.007	0.0007	0.0015	0.0116	0.0034	0.0741	0.0098	0.0045	0.0963	0.0524	1.915	0.4185	0.8607	0.0021	0.1936	0.1644	0.0003	0.0044	0.0156	0.0442	0.0029	0.0012	0.0357	0.0004	NA	0.0083
Zhejiang	0.0214	0.0001	0.0181	0.0166	0.0004	8.8013	0.001	0.0073	0.2066	0.0079	18.9586	0.7054	0	0.0003	1.8066	0.0204	0.0218	0.0006	0.0049	1.2751	0.0001	0.0125	1.0329	0.0001	0.0112	NA

Table S3. Markov Jump Matrix of Global.

	Canada	China	Colombia	Ecuador	Europe	Japan	Korea	Mexico	Philippines	Thailand	USA	Vietnam
Canada	NA	0	0.0032	0	0	0.2825	0.0227	0.026	0	0	5.6104	0
China	0.0032	NA	0.2468	0	2.6786	1.6104	4.8799	0.9091	0	0	13.8864	0.2857
Colombia	0	0.0519	NA	0	0.0032	0.4545	0.1558	0.0032	0	0	9.2143	0
Ecuador	0	0	0	NA	0	0.0487	0.0032	0.0032	0	0	0.9318	0
Europe	0	2.6071	0	0	NA	0.0065	0.013	0.0032	0	0	0.9805	0
Japan	0.0162	0.2727	0.0877	0	0.0097	NA	2.487	0.0487	0	0	53.2695	0.0195
Korea	0	0.9675	0.0162	0	0.026	1.0682	NA	0.0974	0	0	41.974	0.0032
Mexico	0.0032	0.0292	0.0032	0	0	0.2013	0.1169	NA	0	0	13.5065	0
Philippines	0	0.0877	0.039	0	0.0032	0.026	0.0065	0.0032	NA	0	0.8442	0
Thailand	0	0.0779	0.0097	0	0.013	0.4286	0.5909	0.0097	0	NA	0.8539	0
USA	0.0032	2.5942	0.0487	0	0.0292	0.4545	2.2305	0.224	0	0	NA	0.0032
Vietnam	0	6.4675	0.0032	0	0.0422	0.3377	0.1981	0.0097	0	0	2.9805	NA
